# Research Progress on VO_2_-Based Smart Windows: From Phase Transition Mechanisms to Performance Regulation

**DOI:** 10.3390/ma19163523

**Published:** 2026-08-19

**Authors:** Ying Peng, Zhenni Cai, Kecheng Liu, Yuzhuo Ma, Shisong Jin, Pinghua Tang, Haining Ji

**Affiliations:** 1 School of Physics and Optoelectronics, Xiangtan University, Xiangtan 411105, China; 2 Hunan Engineering Laboratory for Microelectronics, Optoelectronics and System on a Chip, Xiangtan University, Xiangtan 411105, China

**Keywords:** vanadium dioxide, smart windows, phase transition mechanisms, performance regulation, research progress

## Abstract

Vanadium dioxide (VO_2_), a typical thermochromic material, undergoes a metal–semiconductor transition (MST) near 68 °C, accompanied by lattice distortion and band structure reconstruction, making it an ideal material for smart window applications. However, VO_2_ faces several bottlenecks that limit its engineering applications, including a relatively high phase transition temperature, poor color comfort, trade-offs between visible light transmittance and solar modulation efficiency, and inadequate long-term stability. This paper first discusses the structural evolution and mechanisms of the VO_2_ phase transition. It then focuses on the three core performance aspects of VO_2_-based smart windows: phase transition temperature regulation, color regulation, and optical performance regulation. Furthermore, it systematically reviews the latest research advancements in key technologies, including elemental doping, interfacial strain engineering, micro- and nanostructure engineering, multilayer film design, and inorganic–organic composite modification. Finally, the paper analyzes current challenges in terms of long-term stability, low-temperature flexible fabrication, skin comfort, and environmental friendliness, and discusses optimization pathways and future prospects for the practical application of VO_2_-based smart windows.

## 1. Introduction

According to statistics from the International Energy Agency (IEA), building energy consumption accounts for more than 30% of total global end-use energy consumption, of which approximately 40% comes from heat exchange through windows and associated air conditioning loads. Consequently, the development of energy-efficient smart windows has become a key focus in building energy conservation [1].

In the 1980s, Svensson and Granqvist [2] proposed the concept of the “smart window,” which integrates optically variable color-changing materials with substrates such as glass to achieve dynamic control of solar radiation and adaptive regulation of indoor temperature. Based on their chromogenic mechanisms and response stimuli [3], smart windows can be classified into photochromic, electrochromic, and thermochromic types. Among these, thermochromic smart windows offer a simple structure and low cost, enabling passive and fully automatic light and heat regulation, and are therefore regarded as an ideal candidate for passive energy-saving smart windows [4].

Vanadium dioxide (VO_2_), a typical thermochromic material, has attracted significant attention since Morin first discovered its metal–semiconductor transition (MST) properties in 1959 [5]. At approximately 68 °C, this material undergoes a rapid reversible phase transition between the monoclinic phase (M1) and the tetragonal rutile phase (R). This transition is accompanied by changes in lattice symmetry and electronic structure, resulting in significant abrupt changes in its optical, electrical, and thermal properties [6]. Due to its comparatively low phase transition temperature relative to other thermochromic materials and its excellent spectral tuning properties, VO_2_ has become a research hotspot in the fields of smart thermal regulation and energy conservation, as well as an ideal material for smart window applications [7,8]. Below the phase transition temperature, VO_2_ exists in the monoclinic insulating phase, exhibiting high transmittance for near-infrared light, which allows solar thermal radiation to enter indoor spaces. Conversely, when transformed into the tetragonal rutile metallic phase, its near-infrared transmittance drops sharply, while visible light transmittance remains largely unchanged [9]. Based on this, VO_2_ thin films can adaptively regulate infrared transmission characteristics in response to the environment: blocking infrared radiation in summer to prevent indoor overheating, and conducting infrared heat in winter for indoor heating. This capability effectively reduces building energy consumption while preserving indoor natural lighting [10].

**Figure 1:**
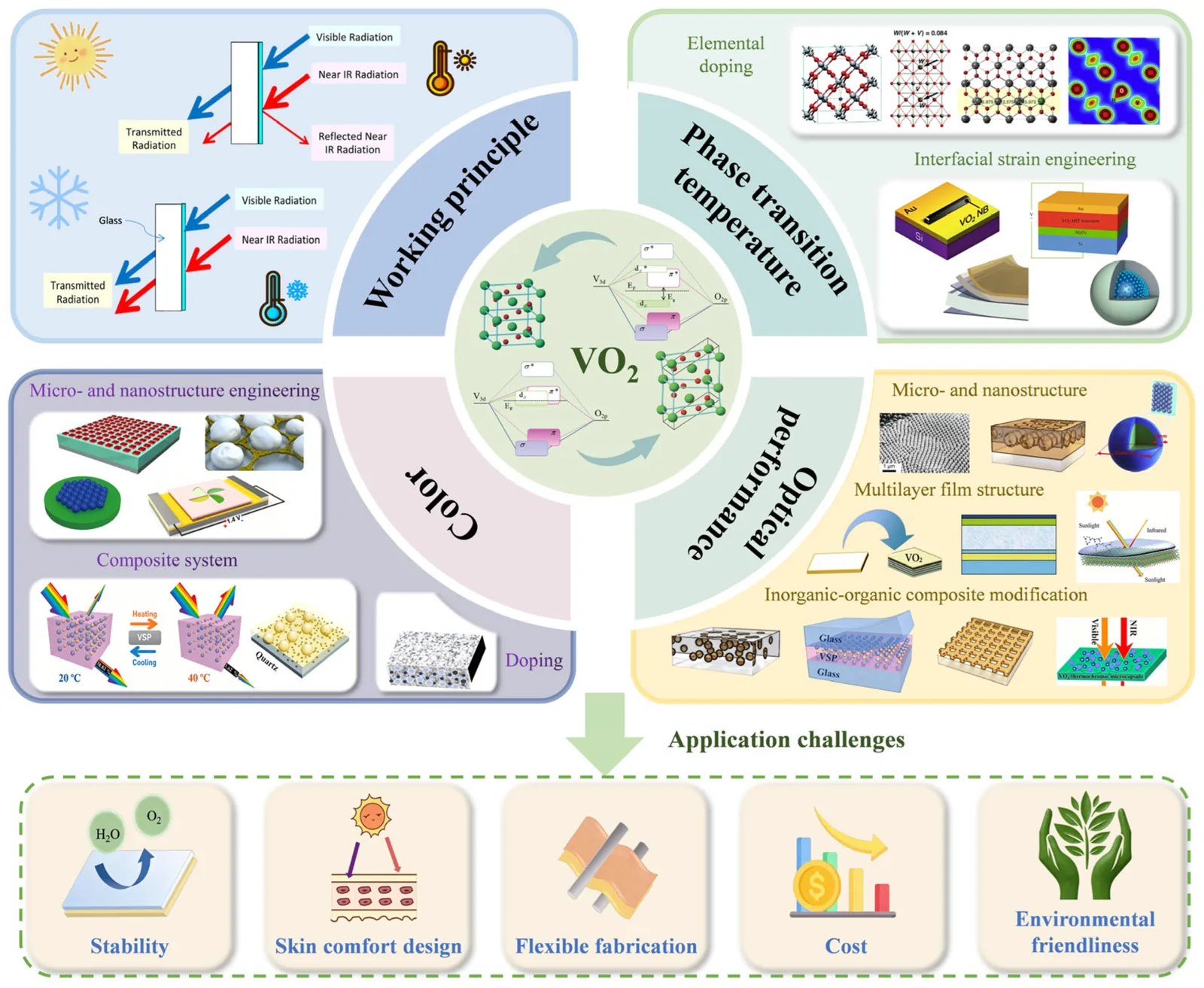
Performance regulation and application challenges of VO_2_-based smart windows. Central schematic: reversible VO_2_ crystal-structure transition between monoclinic M1 and tetragonal R-phase during metal–semiconductor transition (MST). Top-left blue panel: smart window working principle for visible-light and near-infrared adaptive modulation. Top-right light-green panel: phase-transition-temperature tuning via elemental doping and interfacial strain engineering. Right yellow panel: optical performance optimization routes of micro-and-nanostructure fabrication, multilayer film design and inorganic–organic composite modification. Left purple panel: color-tuning strategies. Bottom green dashed box: practical bottlenecks covering stability, indoor thermal comfort, flexible-film fabrication, manufacturing cost and eco-friendly processing.

Recent research on VO_2_-based smart windows has focused on addressing their inherent drawbacks, including high phase transition temperatures, low visible light transmittance, insufficient solar modulation efficiency, undesirable yellowish appearance, and poor stability [11,12]. In response, various performance regulation strategies have been developed, such as elemental doping, multilayer film design, micro- and nanostructure engineering, interfacial strain engineering, and inorganic–organic composite modification [13,14,15]. Meanwhile, as application scenarios continue to expand, increasing attention has been paid to properties such as stability, color neutrality, low-temperature flexible fabrication, skin comfort, and environmental friendliness, driving the development of VO_2_-based smart windows toward high performance, multifunctionality, low cost, and scalable application [16,17]. These optimization strategies and practical bottlenecks are schematically outlined in Figure 1.

Although significant progress has been made in the performance regulation of VO_2_-based smart windows, a comprehensive and systematic review focusing on the regulation of the three core properties, namely phase transition temperature, color, and optical performance, is critically important. This paper first elucidates the structure and phase transition mechanisms of VO_2_, then provides a comprehensive review of the latest research progress in tailoring these three properties, followed by an analysis of the current application status of VO_2_-based smart windows in scenarios such as building energy conservation. Finally, the key technical challenges and future research directions are discussed.

## 2. Structural Transformation and Mechanisms of VO_2_ Phase Transition

### 2.1. Structural Transformation

VO_2_ is a prototypical temperature-controlled phase transition material that undergoes a reversible MST near 68 °C. The physical origin of this phenomenon lies in lattice distortion and concomitant electronic band restructuring. At different temperatures, VO_2_ primarily exists in two stable phases: the low-temperature monoclinic M1 phase and the high-temperature tetragonal rutile R phase. These two phases have attracted the most attention in the research of VO_2_-based smart windows, owing to their comparatively low phase transition temperature relative to other thermochromic materials and the accompanying dramatic changes in optoelectronic properties.

**Figure 2:**
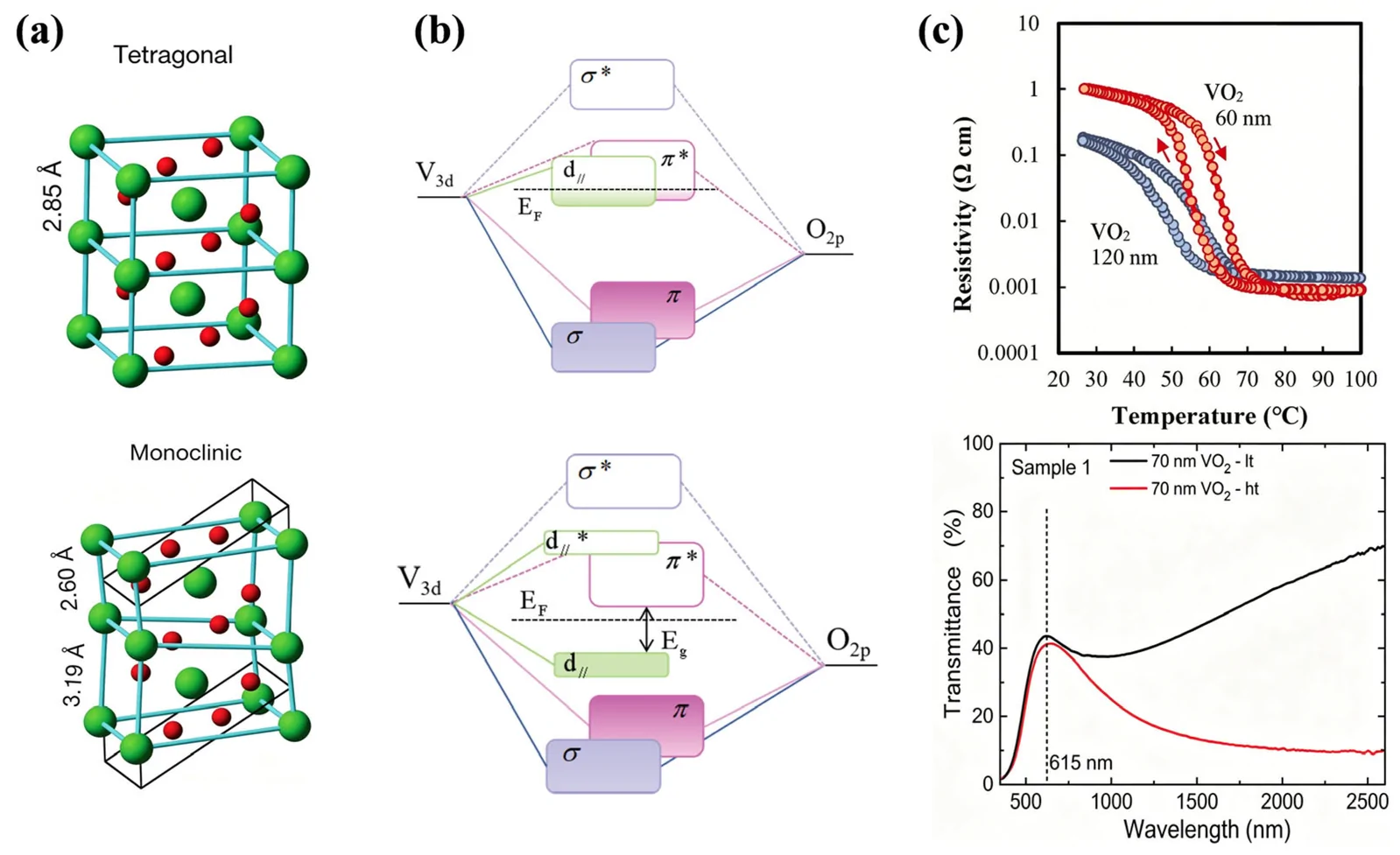
Structural evolution of the VO_2_ phase transition: (**a**) Lattice structures of VO_2_ in the R phase (**top**) and M1 phase (**bottom**), and (**b**) band structure [4], copyright 2015, Royal Society of Chemistry. (**c**) Temperature-dependent resistivity of 120 nm and 60 nm VO_2_ thin films during the phase transition (**top**) [20], copyright 2023. Elsevier and wavelength-dependent transmittance of a 70 nm VO_2_ monolayer film at high and low temperatures (**bottom**) [7], copyright 2019, Royal Society of Chemistry. The right-pointing arrow indicates the heating process, and the left-pointing arrow represents the cooling process.

At temperatures above the critical temperature (T > T_c_), VO_2_ adopts the rutile R phase, which belongs to the tetragonal crystal system (see the upper panel of Figure 2a). Its space group is P4_2_/mnm (No. 136), with lattice parameters a_r_ = b_r_ = 0.455 nm and c_r_ = 0.286 nm. The unit cell angles α, β, and γ are all 90°, indicating a highly symmetric structure [18]. In this phase, V^4+^ ions occupy the vertices and the body-centered position of the tetragonal unit cell, each uniformly coordinated by six O^2−^ ions, forming a regular VO_6_ octahedron. Along the z-axis, the interatomic distance between adjacent V atoms is 0.287 nm, resulting in a nearly equidistant chain-like arrangement. This unobstructed electron delocalization pathway imparts metallic conductivity to the material [19].

When the temperature drops below the phase transition temperature (T < T_c_), VO_2_ transforms into the M1 phase, which belongs to the monoclinic crystal system (see the lower half of Figure 2a). The space group reduces to P2_1_/c (No. 14), the unit cell parameters change to a_m_ = 0.575 nm, b_m_ = 0.453 nm, and c_m_ = 0.538 nm, and the β angle increases to 122.6°, resulting in a significant reduction in overall symmetry [20,21].

According to the classic study by Morin et al., the core of this transformation involves the paired displacement (dimerization) of V atoms along the a-axis of the crystal, forming two V–V bonds of different lengths: a short bond of approximately 0.265 nm and a long bond of approximately 0.312 nm, with the V atoms arranged in a zigzag (Z-shaped) alternating pattern [22,23]. Concurrently, the VO_6_ octahedra undergo tilting distortion, transforming from regular octahedra to semi-octahedra, accompanied by a unit cell volume change of approximately 0.044%, which further exacerbates the degree of lattice distortion [24]. This V atom dimerization and octahedral distortion directly lead to the localization of electrons in the vanadium 3d orbitals, thereby blocking the electron delocalization pathways and driving the transition of VO_2_ from a metallic to an insulating state. It is worth noting that near the phase transition temperature between the M1 and R phases, VO_2_ is typically accompanied by the appearance of intermediate metastable phases such as the M2 and T phases. The existence of these intermediate structures confirms that the phase transition is not strictly a one-step process but exhibits distinct gradual characteristics. However, since this paper focuses on the steady-state structure and performance regulation for smart window applications, the fine structure of the intermediate phases will not be explored in depth.

In addition to lattice distortion, VO_2_ undergoes a transformation in its electronic band structure near the Fermi level during the MST. Figure 2b clearly compares the differences in the band structure of VO_2_ at high and low temperatures, intuitively illustrating the core transformation mechanisms of orbital hybridization and electronic state localization.

According to the classical orbital model established by Goodenough et al. [25], in the high-temperature rutile R phase, the 3d orbitals of V^4+^ ions split under the influence of the VO_6_ octahedral crystal field, forming a lower-energy t_2g_ state and a higher-energy e^σ^_g_ state. Specifically, the t_2g_ orbital further splits into π and π* orbitals, as well as d_//_ orbitals distributed along the c-axis; the e^σ^_g_ orbital splits into a bonding σ orbital and an antibonding σ* orbital. The aforementioned π- and σ-orbitals can further hybridize with the oxygen 2p orbital, forming two distinct energy bands: a lower-energy bonding state dominated by the O_2p_ orbital, and a higher-energy antibonding state dominated by the V_3d_ orbital. In the highly symmetric R phase, the d_//_ energy band distributed along the c-axis overlaps with the π* antibonding energy band. The Fermi level traverses this overlapping region, resulting in metallic conductivity in VO_2_.

When the temperature drops below the phase transition temperature, the dimerization of V atoms and the accompanying lattice distortion trigger a dramatic reorganization of the electronic states, driving VO_2_ into the monoclinic M1 phase. Research by Budai et al. [26] indicates that the formation of V–V dimers significantly alters the interorbital interactions, causing the d_//_ orbitals—which originally overlapped in the metallic state—to split into a low-energy, fully occupied d_//_ band and a high-energy, empty d_//_* band. Simultaneously, the π* antibonding orbitals, which previously overlapped with the d_//_ band, shift toward higher energies, creating a bandgap of approximately 0.7 eV between the d_//_ and π* orbitals [27]. At this point, the Fermi level lies at the center of this bandgap. With no free electrons available to be excited into the conduction band and the delocalized electron transport pathway blocked, VO_2_ exhibits typical insulating behavior.

As can be seen from the above, the lattice distortion and band restructuring induced by the phase transition cause VO_2_ to exhibit temperature-dependent optoelectronic properties. Its MST and transmission spectra are shown in Figure 2c, which forms the basis for its adaptive regulation in smart windows. Note that the resistivity magnitude of VO_2_ thin films over the metal–semiconductor transition is strongly governed by film thickness, crystallinity, intrinsic defects and fabrication parameters. Its resistivity can vary from megaohms down to a fraction of an ohm or even lower. The plotted curves are merely representative experimental data for particular VO_2_ specimens.

### 2.2. Phase Transition Mechanisms

Currently, three main representative viewpoints have been proposed regarding the nature of the phase transition in VO_2_: the Peierls phase transition driven by changes in the lattice structure, the Mott–Hubbard phase transition driven by strong electron correlations, and the synergistic mechanism involving both effects.

#### 2.2.1. Peierls Phase Transition Mechanism

**Figure 3:**
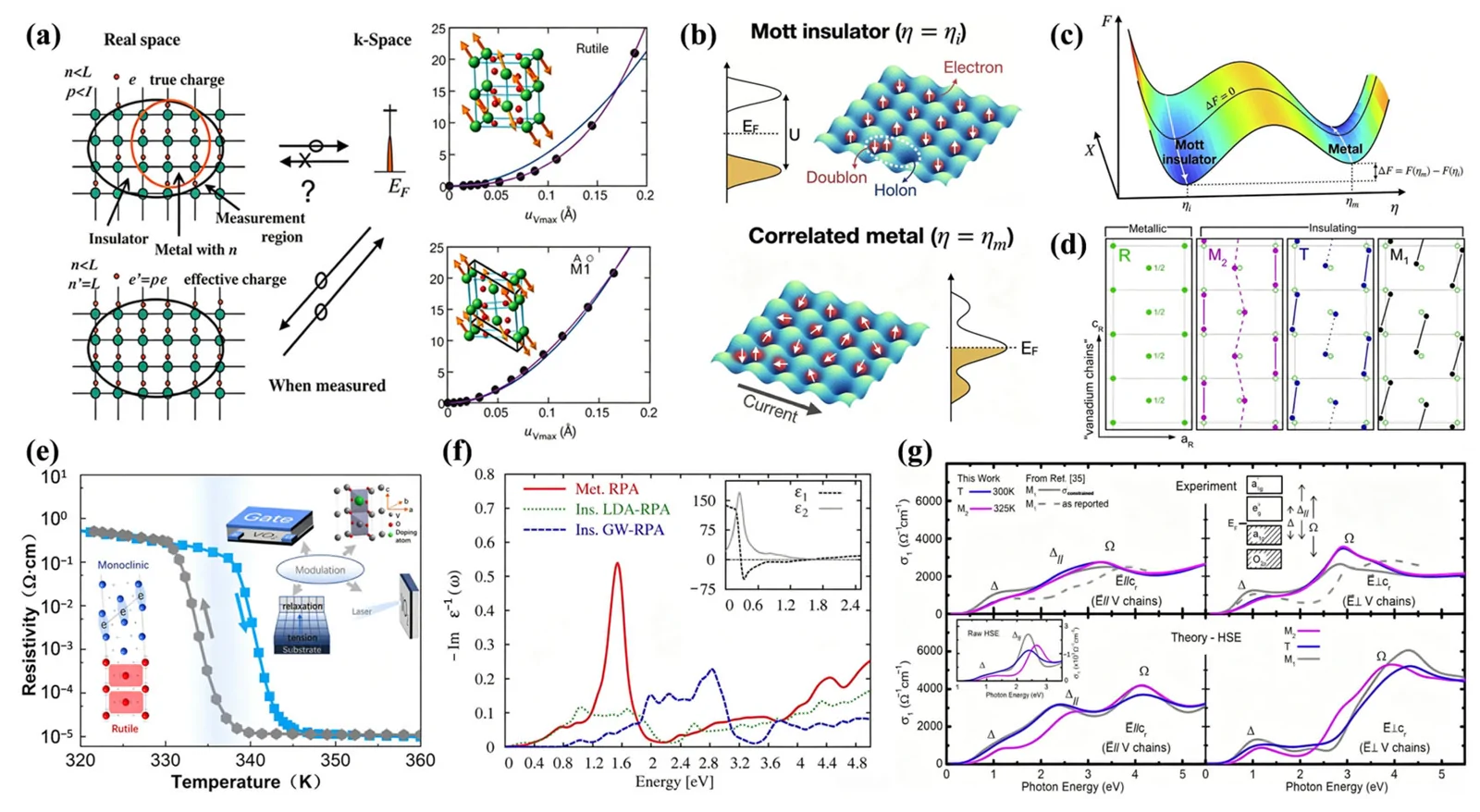
Investigation of the VO_2_ phase transition mechanism: (**a**) Schematic diagrams of the Peierls and Mott phase transitions [29], copyright 2004, IOP Publishing. The orange-colored circle highlights the local metallic domain embedded in the insulating background; the question mark indicates the ambiguous mapping relationship from this real-space inhomogeneity to the k-space electronic-structure; the orange arrows in the crystal-structure inset denote the displacement vectors of vanadium atoms induced by lattice distortion. (**b**) Band structures of the Mott semiconductor phase (**top**) and metal phase (**bottom**) in the single-band Hubbard model, where the single-particle energy spectrum of the Mott semiconductor splits into upper and lower Hubbard bands, and the bandgap width is comparable to the on-site Coulomb repulsion energy U. (**c**) Curves showing the variation of the system’s free energy functional F with respect to system variables η (electron configuration and lattice configuration) and control parameter X (applied voltage or carrier density of specific holes) under local thermal equilibrium [34], copyright 2024, Springer Nature. The color gradient on the free-energy surface visualizes the magnitude of free-energy values, where darker-blue regions correspond to low-energy stable states and warm-toned colors represent higher free-energy barriers. (**d**) Vanadium ion arrangement structures in different crystal phases of VO_2_ [35], copyright 2017, American Physical Society. (**e**) The phase transition process and thermal hysteresis characteristics of specific VO_2_ thin-film sample [40], copyright 2018, Springer Nature; the resistivity range of VO_2_ films during MST is affected by multiple preparation-related factors. The upward gray arrow and the downward blue arrow represent the heating branch and cooling branch of the hysteresis loop during the metal-insulator transition, respectively. (**f**) Comparison of energy loss functions (EELS) for the metallic and insulating phases calculated using the GW-RPA and LDA-RPA methods, respectively. The plasma peak at 1.5 eV observed in the metallic phase disappears in the insulating phase, consistent with experimental observations [41], copyright 2021, Elsevier. (**g**) Optical conductivity curves for the T phase and M2 phase, where neither the electronic structure nor the bandgap of the two phases has changed, and the experimental data agree with the HSE theoretical calculations [35], copyright 2017, American Physical Society.

In the 1930s, Peierls proposed that, from a lattice structure perspective, a one-dimensional equidistant metal chain spontaneously undergoes structural distortion at low temperatures. Through atomic dimerization, the lattice period doubles, thereby opening a bandgap at the Fermi level and achieving a transition from a metal to a semiconductor (see Figure 3a) [28,29].

Numerous studies support the view that the VO_2_ phase transition is a Peierls phase transition from various perspectives. For example, Liebsch et al. [30] found through quasiparticle spectroscopy that the t_2g_ orbitals in the VO_2_ (R) phase exhibit extremely weak polarization but strong dynamic correlations, whereas in the M1 phase, the 3d orbitals show strong polarization and pronounced V–V Peierls distortion, which is a key factor driving the transition to the insulating state. Budai et al. [26] utilized X-ray and neutron scattering techniques to discover non-harmonic phonon effects during the VO_2_ phase transition, confirming the decisive role of lattice distortion in the process. Gervais et al. [31], through analysis of the phonon dispersion curve, pointed out that the instability of the VO_2_ lattice is the fundamental cause of the MST, and the phonon softening characteristics corroborate the structural driving mechanism of Peierls distortion. Furthermore, Cavalleri et al. [32] used ultrafast spectroscopy to observe that the low-temperature semiconductor phase of VO_2_ exhibits prominent band-like characteristics, which are caused by Peierls-type lattice distortion, thereby demonstrating that the MST in VO_2_ is primarily structure-driven.

#### 2.2.2. Mott–Hubbard Phase Transition Mechanism

In contrast, the Mott–Hubbard mechanism emphasizes strong electron correlations, positing that the MST in VO_2_ is governed primarily by electron–electron interactions rather than by structural changes. According to Mott theory, when the interelectron distance decreases to the critical threshold n_e_^1/3^a_H_ ≈ 0.2 (where a_H_ is the Bohr radius of the valence electrons), the upper and lower Hubbard bands overlap. Under these conditions, kinetic energy dominates, and the system exhibits metallic behavior. Conversely, when electron localization intensifies and Coulomb repulsion prevails, the two bands separate completely, driving the system into an insulating state (see Figure 3a–d) [29,33,34,35].

Laad et al. [36] employed a method combining the Local Density Approximation (LDA) with the Dynamical Mean-Field Theory (DMFT) to investigate the evolution of electronic orbitals during the VO_2_ phase transition. The computational results showed semi-quantitative agreement with experimental data from polarized X-ray absorption spectroscopy, indicating that the VO_2_ phase transition is a Mott–Hubbard phase transition. Furthermore, Petrov et al. [37] found through Raman spectroscopy analysis that the evolution of phonon vibration modes in VO_2_ near the phase transition temperature is primarily dominated by strong electron correlations, i.e., a Mott–Hubbard phase transition. Kim et al. [38] confirmed via femtosecond pump–probe techniques that the structural phase transition and MST in VO_2_ do not occur simultaneously: the MST begins at 54 °C, with the electronic state already exhibiting metallization features, while lattice distortion is not completed until higher temperatures. Using scanning near-field infrared microscopy, Qazilbash et al. [39] directly observed the nucleation and growth of metallic “puddles” during the VO_2_ phase transition. The scattering amplitude of the metallic nanoclusters was 2–5 times greater than that of the insulating phase. This pronounced contrast originates from strong electronic correlations in the metallic state.

#### 2.2.3. Synergistic Mechanism

However, both the Peierls phase transition theory and the Mott–Hubbard phase transition theory have their limitations, making it difficult to explain all experimental phenomena observed during the VO_2_ phase transition using a single theoretical model. A growing body of experimental evidence suggests that the MST of VO_2_ is not governed by a single mechanism, but rather results from the synergy between the Peierls phase transition and the Mott–Hubbard phase transition (see Figure 3e–g) [35,40,41].

First, Koethe et al. [42] studied the valence and conduction bands of VO_2_ using photoelectron spectroscopy and X-ray absorption spectroscopy, finding that the drastic changes in the band structure during the phase transition could not be explained by either the Mott or Peierls theory alone. Subsequently, Yao et al. [43] combined synchrotron temperature-dependent X-ray absorption fine structure spectroscopy with density functional theory and found that both the V–V bond angle and electrical resistance exhibited significant hysteresis loops during the VO_2_ phase transition, leading them to conclude that this is the result of the synergistic interaction between lattice structure transformation and electron correlations. Furthermore, Tao et al. [44] utilized femtosecond time-resolved electron diffraction to reveal a two-stage kinetics in the VO_2_ phase transition: an instantaneous transition of the electronic state on the sub-picosecond timescale, followed by a structural reorganization dominated by lattice instability over approximately 10 ps, thereby elucidating a coupling mechanism initially triggered by electron correlations and synergistically driven by Peierls distortions.

Currently, the mechanism underlying the VO_2_ phase transition remains a subject of debate, as all three theories have garnered support from different experimental observations. The prevailing view holds that this phase transition most likely arises from a synergistic interplay between electron correlations and lattice distortion. No single mechanism can fully account for the diverse experimental observations reported to date. The dominant mechanism likely depends on the specific external stimuli (such as optical or electric fields) and the material synthesis route (including epitaxial strain and defect engineering).

## 3. Phase Transition Temperature Regulation

The intrinsic phase transition temperature (T_c_) of VO_2_ is approximately 68 °C, which is significantly higher than the indoor comfort temperature range in buildings. Consequently, VO_2_ in its pristine form cannot directly meet the requirements for energy-saving window applications. To address this issue, researchers have primarily conducted studies on phase transition temperature regulation through elemental doping and interfacial strain engineering. Representative results are summarized in Table 1.

### 3.1. Elemental Doping

Early studies primarily employed single-element doping to regulate the phase transition behavior of VO_2_. Based on the valence state differences of dopant ions, a clear regulatory mechanism has emerged in the academic community: high-valent cations (such as Nb^5+^, Mo^6+^, and W^6+^ [45,46,47]) can inject free electrons into the VO_2_ lattice, which reduces the splitting gap of the V_3d_ orbitals and weakens V–V dimerization, thereby significantly lowering T_c_; whereas low-valent cations (such as Ti^4+^, Al^3+^, Cr^3+^, etc. [48,49,50]) exacerbate lattice distortion and enhance electron localization effects, leading to an increase in T_c_.

Among the various dopants capable of lowering T_c_, W doping has emerged as a prominent strategy owing to its exceptional modulation efficiency. To quantitatively assess the doping effect, the phase transition temperature reduction efficiency R was introduced as a key figure of merit. Ji et al. [51] performed the first quantitative comparison of the temperature-lowering efficiency of various dopants. W_x_V_1−x_O_2_ nanopowders were synthesized via a hydrothermal route, and combined DSC analysis and computational fitting revealed a reduction efficiency R of 21.96 °C/at% for W doping, substantially surpassing that of Mo and Nb and highlighting the distinct superiority of tungsten. Subsequent efforts extended this work by exploring the influence of core–shell architectures and annealing treatments on the overall performance of W-doped systems. The modulation efficiency of different synthesis routes, however, varied considerably. Zhou et al. [52] integrated the hydrothermal phase method with a microemulsion approach to fabricate W_x_V_1−x_O_2_@SiO_2_ core–shell nanomaterials, achieving an optimized R of 21.27 °C/at% along with markedly enhanced stability. Zhu et al. [53] applied post-annealing to hydrothermally synthesized W_x_V_1−x_O_2_@SiO_2_ nanopowders, which reduced R to 14.5 °C/at%. At a W doping level of 2%, T_c_ was lowered to 40.4 °C with good optical performance retained. To balance phase transition temperature regulation with practical device performance, researchers have also incorporated W-doped VO_2_ powders into polymer matrices, creating functional composite films with improved overall properties. Yang et al. [54] employed ammonium metavanadate as the vanadium precursor, hydrazine hydrate as the reducing agent, and ammonium metatungstate as the W source in a one-step hydrothermal synthesis, producing VO_2_ powders with doping levels from 0 to 2.0 at%. These powders were subsequently embedded into a polyurethane (PU) matrix to form VO_2_/PU composite films. DSC measurements showed that as the W content increased from 0 to 2.0 at%, the heating-cycle T_c_ decreased linearly from 61.8 °C to 15 °C, corresponding to a fitted R of ~18.42 °C/at% and demonstrating remarkably efficient temperature-lowering regulation. The composite film based on 1.0 at% W-doped VO_2_ powder delivered a solar modulation efficiency of 11.7%, substantially lowering the phase transition temperature while satisfying the essential requirements for smart window applications. Notably, Calvi et al. [55] reported an outstanding R of 24.5 °C/at% for W-doped VO_2_ via high-temperature annealing of vanadyl oxalate precursor, which represents the best R-value documented prior to 2021. Subsequent research based on pulsed-laser-deposited (PLD) thin-film systems further lifted the performance ceiling of W-doping modulation. Bleu et al. [56] prepared W-doped VO_2_ thin films by PLD of V/W (+O_2_) and V_2_O_5_/W multilayers and subsequent 400–450 °C rapid-thermal annealing under oxygen flow. A high R of −26 °C per at.% W was achieved, and the 1.7 at.% W-doped sample realized T_c_ at 26 °C with 62% luminous transmittance for smart window applications.

Despite these advances, single-element doping suffers from inherent drawbacks. High doping concentrations tend to introduce excessive lattice defects, reduce visible light transmittance, and broaden the phase transition hysteresis [57,58]. Multi-element co-doping has therefore emerged as a promising strategy, leveraging the complementary functions of different ions to achieve synergistic gains in temperature reduction, phase stability, visible light transmittance, and hysteresis narrowing. Extensive efforts have been devoted to metal co-doping systems, with researchers exploring a wide range of cation combinations and processing routes. Co-doping with light metals (e.g., Mg, Zn) alongside W or Mo has been shown to enhance visible light transmittance and suppress the yellow tint without compromising the temperature-lowering effect [59]. Kang et al. [60] fabricated W-Zr co-doped VO_2_ films via magnetron sputtering. At doping levels of 0.95 at% W and 0.3 at% Zr, the visible light transmittance rose from 26.2% to 48.4% and T_c_ dropped to 36 °C. Zhang et al. [61] synthesized W-Mo co-doped VO_2_ films by a sol–gel route, demonstrating that both Mo and W substitute for V in the lattice. Following co-doping, T_c_ decreased, the hysteresis width narrowed, and the phase transition temperature window broadened. Additionally, Li et al. [62] prepared W-Al co-doped VO_2_ nanoparticles and thin films using a sol–gel method followed by annealing. The synergistic interaction between W and Al reduced T_c_ to 43.8 °C and narrowed the hysteresis width to 7.5 °C, while markedly enhancing the oxidation stability of the films. This work offers a promising strategy for near-room-temperature applications.

In parallel, non-metallic element doping has advanced rapidly as a low-damage, high-transparency alternative, serving as an important complement to metal doping. Elements such as N, B, S, and Se can substitute oxygen sites or occupy interstitial positions, gently modulating the carrier concentration and lattice strain. This effectively alleviates the severe lattice distortion typically induced by metal doping. Experimentally, Kano et al. [63] demonstrated efficient nitrogen incorporation into VO_2_ thin films via mist chemical vapor deposition, achieving a T_c_ of approximately 29.5 °C. This result further validates non-metallic doping as an effective strategy for phase transition temperature regulation in smart window applications. Complementary theoretical investigations further indicate that low-concentration doping with chalcogen elements (e.g., S, Se) can deliver a temperature reduction efficiency of ~35 K/at% while preserving excellent infrared modulation contrast, underscoring its considerable application potential [64].

### 3.2. Interfacial Strain Engineering

Interfacial strain engineering represents another key method for regulating the phase transition temperature of VO_2_. Beyond external bending or tensile forces that induce macroscopic strain, interfacial strain is primarily generated during film growth through lattice mismatch, the introduction of buffer layers, or the coupling of multilayer films and core–shell structures. This approach creates a controllable stress field within the VO_2_ lattice, thereby lowering T_c_. Importantly, this method introduces no impurities, effectively preserving the intrinsic optical performance of VO_2_, and mitigates issues such as reduced visible light transmittance and yellowish coloration. Consequently, interfacial strain engineering is particularly attractive for high-performance smart window applications.

**Figure 4:**
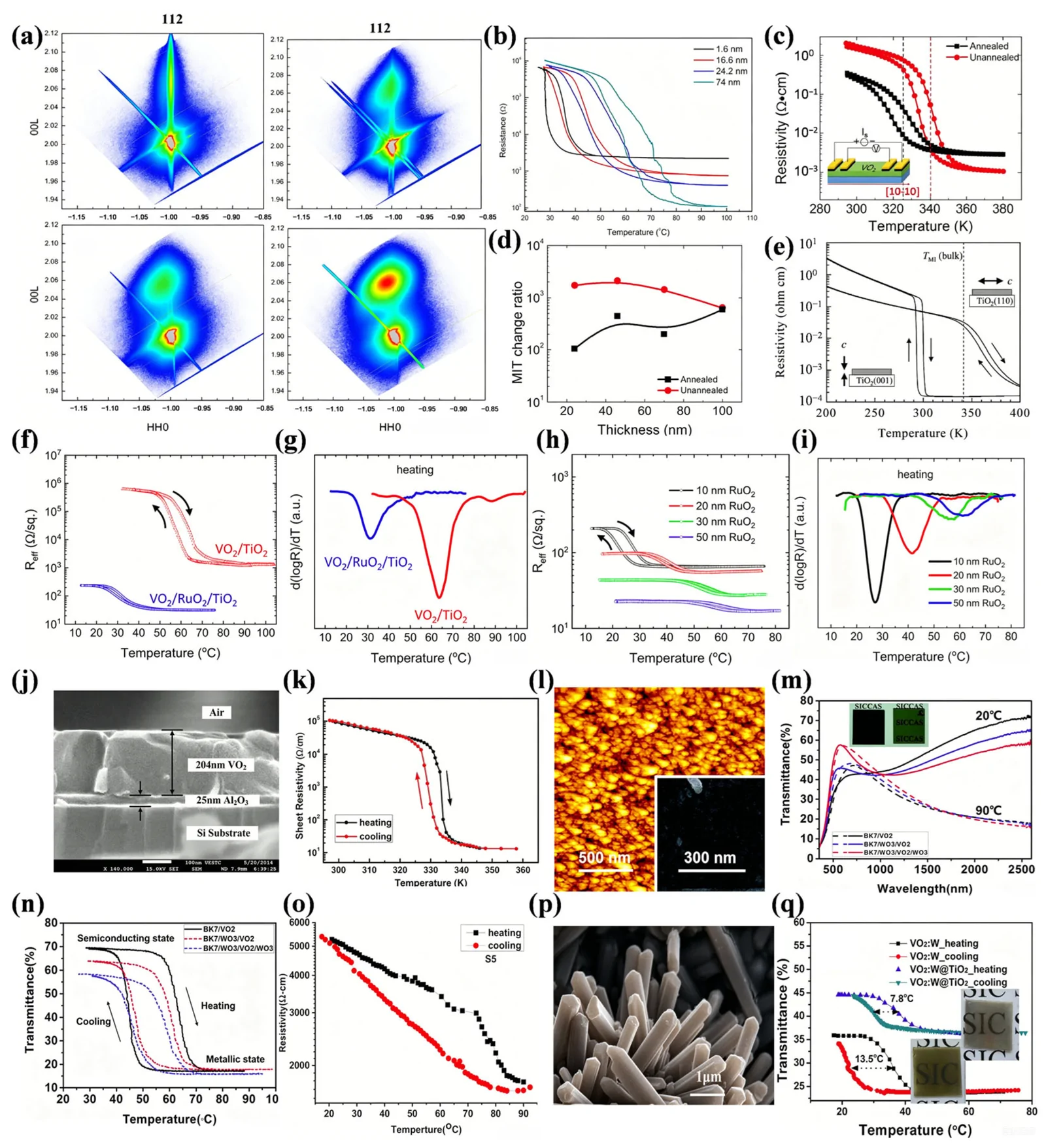
VO_2_ interfacial strain engineering: (**a**) Reciprocal space maps of VO_2_ films on TiO_2_ substrates of different thicknesses [65], copyright 2014, American Chemical Society. The color brightness in the reciprocal-space intensity map represents the magnitude of diffraction intensity, where brighter (warmer-toned) colors correspond to higher diffraction intensity. (**b**) Resistance–temperature curves of VO_2_ films with different thicknesses [65], copyright 2014, American Chemical Society. (**c**) Resistivity–temperature curves of 24 nm-thick VO_2_ films on annealed and unannealed substrates, and (**d**) the MST variation ratio of VO_2_ films under different thickness conditions [66], copyright 2023, Elsevier. (**e**) Resistivity–temperature curves of VO_2_ films on TiO_2_ substrates with different crystal orientations [67], copyright 2002, American Institute of Physics. The arrows indicate the heating and cooling directions during the phase-transition process. (**f**) Comparison of the sheet resistance–temperature characteristics of 100 nm-thick VO_2_ films on TiO_2_ substrates with and without a RuO_2_ buffer layer, along with their (**g**) differential curves [69], copyright 2019, American Institute of Physics. The black arrows represent the heating-cooling cycle direction of the hysteresis loop. (**h**) The sheet resistance–temperature curves of 50 nm-thick VO_2_ films grown on TiO_2_ substrates with different RuO_2_ buffer layer thicknesses (10–50 nm) and their (**i**) differential curves [69], copyright 2019, American Institute of Physics. The black arrows mark the heating and cooling directions of the resistance hysteresis. (**j**) Cross-sectional SEM images of Si/Al_2_O_3_/VO_2_ films and (**k**) their sheet resistance–temperature curves [70], copyright 2014, Elsevier. (**l**) SEM image (bottom right) and AFM image (remaining portions) of a WO_3_/VO_2_/WO_3_ film [71], copyright 2016, Royal Society of Chemistry. (**m**) Comparison of transmission spectra and optical photographs (top left) of WO_3_/VO_2_/WO_3_ films versus other films at different temperatures, as well as (**n**) the transmittance–temperature curve [71], copyright 2016, Royal Society of Chemistry. (**o**) Resistivity–temperature curves of Mo_0.2_W_0.8_O_3_/VO_2_/MoO_3_ multilayer films [72], copyright 2022, MDPI. (**p**) SEM image of VO_2_@TiO_2_ core–shell nanorods [74], copyright 2013, Springer Nature. (**q**) Temperature-dependent optical transmittance of W-doped VO_2_ and W-doped VO_2_@TiO_2_ films [74], copyright 2013, Springer Nature. Compared to the pristine W-doped VO_2_ film, the W-doped VO_2_@TiO_2_ bilayer film exhibits a significantly reduced thermal hysteresis width (7.8 °C vs. 13.5 °C) and a higher visible light transmittance across the entire measured temperature range.

Epitaxial growth of VO_2_ films on hetero-substrates such as TiO_2_ and Al_2_O_3_ introduces in-plane stress via lattice mismatch, which in turn modifies the unit cell parameters and the phase transition temperature. Systematic investigations have explored how substrate choice, pretreatment conditions, and crystallographic orientation modulate the substrate-induced stress and the resulting device performance. Fan et al. [65] deposited VO_2_ films on rutile TiO_2_ (001) substrates. Reciprocal space mapping (Figure 4a) revealed that lattice mismatch-induced interfacial stress modifies the V^4+^–V^4+^ spacing, leading to corresponding changes in T_c_. The resistance–temperature characteristics of these films are presented in Figure 4b. Substrate annealing provides an additional means of tuning the in-plane stress. Yang et al. [66] grew VO_2_ films on annealed (0001)-Al_2_O_3_ single-crystal substrates and observed a T_c_ reduction of 14.4 K relative to films on unannealed substrates. The corresponding resistivity–temperature curves are shown in Figure 4c. These results confirm that in-plane compressive stress from lattice mismatch can effectively reduce T_c_ while preserving a moderate MST variation ratio (Figure 4d). Furthermore, the crystallographic orientation of the substrate also plays a critical role, producing marked differences in both the magnitude of lattice mismatch and the nature of the resulting stress. Muraoka and Hiroi [67] systematically compared VO_2_ films grown on TiO_2_ substrates of different orientations. On TiO_2_ (001), the in-plane tensile stress compresses the c-axis, lowering T_c_ to 300 K. In contrast, TiO_2_ (110) induces a larger lattice mismatch, generating stress that elongates the c-axis and raises T_c_ to 369 K. The resistivity–temperature characteristics for both orientations are presented in Figure 4e.

The insertion of buffer layers (e.g., Al_2_O_3_, TiO_2_) between VO_2_ and the substrate provides an effective route to tailor interfacial stress. This modifies the V^4+^–V^4+^ spacing and orbital overlap, thereby tuning T_c_ while simultaneously improving the film’s crystallinity and hysteresis characteristics. Guided by this principle, researchers have explored a variety of buffer layer materials. Zhu et al. [68] deposited a ZnO buffer layer on glass and prepared VO_2_/ZnO composite films via magnetron sputtering. The interfacial compressive strain reduced T_c_ to 48 °C while preserving good optical transmittance. The buffer layer thickness is another critical parameter governing the phase transition behavior. Kim et al. [69] systematically varied the thickness of RuO_2_ buffer layers (10–50 nm) on TiO_2_ (001) substrates. As the buffer thickness decreased, T_c_ dropped continuously from 59 °C to 24 °C. The sheet resistance–temperature characteristics and corresponding differential curves are displayed in Figure 4f–i. For silicon-based platforms, Xiong et al. [70] addressed the lattice mismatch problem by introducing an Al_2_O_3_ buffer layer with optimized oxygen partial pressure. A cross-sectional micrograph of the resulting structure is shown in Figure 4j. This strategy effectively alleviated the lattice mismatch, transforming the VO_2_ grains from a small and irregular morphology into a dense, uniform microstructure. The improved crystallinity lowered the phase transition barrier, thereby reducing T_c_ (Figure 4k), and simultaneously narrowed the hysteresis width to just 4 K. Furthermore, it suppressed the formation of parasitic phases such as V_6_O_13_ and promoted preferential (011) orientation.

Multilayer films and core–shell structures exploit several interfacial synergies to generate a composite stress field. These include interlayer and core–shell lattice mismatch, strain transfer, and confinement effects. The resulting stress field lowers T_c_ while simultaneously enhancing optical performance and stability. Long et al. [71] fabricated a symmetric WO_3_/VO_2_/WO_3_ sandwich structure via reactive magnetron sputtering. AFM and SEM images of the trilayer are presented in Figure 4l. In this design, the bottom WO_3_ layer functions as a buffer, while the top WO_3_ layer serves as an anti-reflection coating. The structure reduced T_c_ to 52 °C with enhanced optical performance. The corresponding transmission spectra and thermal hysteresis loops are shown in Figure 4m,n. Basyooni et al. [72] deposited Mo_0.2_W_0.8_O_3_/VO_2_/MoO_3_ multilayers by high-vacuum sputtering. The combined effects of interfacial strain, oxygen vacancies, and tungsten doping reduced T_c_ to 20 °C, offering a promising route toward near-room-temperature phase transition regulation. The corresponding MST curve is shown in Figure 4o. Core–shell architectures offer another versatile platform for property tuning. The VO_2_@ZnO core–shell system developed by Chen et al. [73] exploits core–shell lattice mismatch to reduce T_c_, while simultaneously boosting visible light transmittance by 31.1% and solar modulation efficiency by 11.0%. Notably, the film retained 77% of its solar modulation efficiency after 1000 h of exposure to high-temperature, high-humidity conditions. Li et al. [74] reported W-doped VO_2_@TiO_2_ core–shell nanorods that further reduced T_c_ to the range of 20–30 °C. The SEM image of these nanorods is presented in Figure 4p. This strategy simultaneously enhances visible light transmittance and stability, and shifts the film color from yellow to light blue, offering valuable insights for multifunctional smart coating design. The temperature-dependent transmittance is shown in Figure 4q. Furthermore, Balin et al. [75] prepared hemispherical Au/VO_2_ core–shell particles. Increasing the Au core size tunes the rutile-phase plasmon resonance from 600 to 720 nm, as confirmed by simulations. Plasmon induction and percolation effects reduce T_c_ by 10 °C, and the transition shift only occurs under optical excitation. Plasmon coupling prevails under high irradiation, while nanoparticle surface thermal strain dominates at regular illumination.

**Table 1:** Regulation of phase transition temperatures in VO_2_-based smart windows.

Authors	Methods	T_c_	Descriptions	Ref.
Guo et al.	W-Sn-Zr co-doping	27.6 °C	W-Sn-Zr co-doped VO_2_ films prepared via the sol–gel method, with significantly improved optical performance.	[12]
Wang et al.	W-Mg co-doping	35 °C	Preparation of W-Mg co-doped VO_2_ films, achieving a decrease in T_c_ and attenuation of the yellow hue.	[45]
Zhu et al.	W doping	40.4 °C	Hydrothermal synthesis of W_x_V_1−x_O_2_@SiO_2_ powder; phase transition characteristics and stability improved after annealing.	[53]
Calvi et al.	W doping	R = 24.5 °C/at%	Tungsten-doped VO_2_ prepared by high-temperature-annealed vanadyl oxalate precursor.	[55]
Bleu et al.	W doping	26 °C R ≈ −26 °C/at%	W-doped VO_2_ thin-film prepared by pulsed laser deposition and rapid-thermal annealing.	[56]
Kang et al.	W-Zr co-doping	36 °C	Magnetron sputtering of W-Zr co-doped VO_2_ films, resulting in increased visible light transmittance.	[60]
Li et al.	W-Al co-doping	43.8 °C	Sol–gel synthesis of W-Al co-doped VO_2_ films, resulting in narrowed phase transition hysteresis and improved stability.	[62]
Kano et al.	N doping	~29.5 °C	N-doped VO_2_ thin films grown on an SnO_2_ buffer layer via mist CVD.	[63]
Daniel et al.	Chalcogen doping	25–30 °C R ≈ −35 K/at%	S/Se-doped VO_2_ films maintain significant optical contrast even at low concentrations.	[64]
Fan et al.	N-S co-doping	Near room temperature	Preparation of N-S co-doped VO_2_ films via annealing, with visible light transmittance increased by 24.3%.	[65]
Yang et al.	Interfacial strain engineering	↓ 14.4 K	VO_2_ films epitaxially grown on annealed Al_2_O_3_ substrates, with T_c_ reduced by 14.4 K compared to unannealed substrates, while maintaining a moderate change in MST.	[66]
Zhu et al.	Interfacial strain engineering	48 °C	Magnetron sputtering of VO_2_/ZnO composite films, while maintaining good optical transmittance.	[68]
Kim et al.	Interfacial strain engineering	24–59 °C	Introduction of a RuO_2_ buffer layer on a TiO_2_ (001) substrate; T_c_ is continuously tunable over a wide range of 24–59 °C by controlling the buffer layer thickness.	[69]
Long et al.	Interfacial strain engineering	52 °C	Magnetron sputtering of WO_3_/VO_2_/WO_3_ multilayer films, with visible light transmittance increased to 55.4% and significantly enhanced stability.	[71]
Basyooni et al.	Interfacial strain engineering	20 °C	Mo_0.2_W_0.8_O_3_/VO_2_/MoO_3_ multilayers prepared by high-vacuum sputtering, with T_c_ controlled by the synergistic regulation of multiple factors.	[72]
Balin et al.	Interfacial strain engineering	↓ 10 °C	Hemispherical Au-VO_2_ core–shell nanoparticles.	[75]

## 4. Color Regulation

VO_2_ exhibits an inherent brownish-yellow coloration, with no perceptible visual difference before and after the phase transition, making it difficult to meet the practical requirements of smart windows. Currently, three main types of strategies have been employed for color regulation: doping, micro- and nanostructure engineering, and composite formation. Based on representative studies, this paper analyzes each of these approaches, with relevant information summarized in Table 2.

### 4.1. Elemental Doping

**Figure 5:**
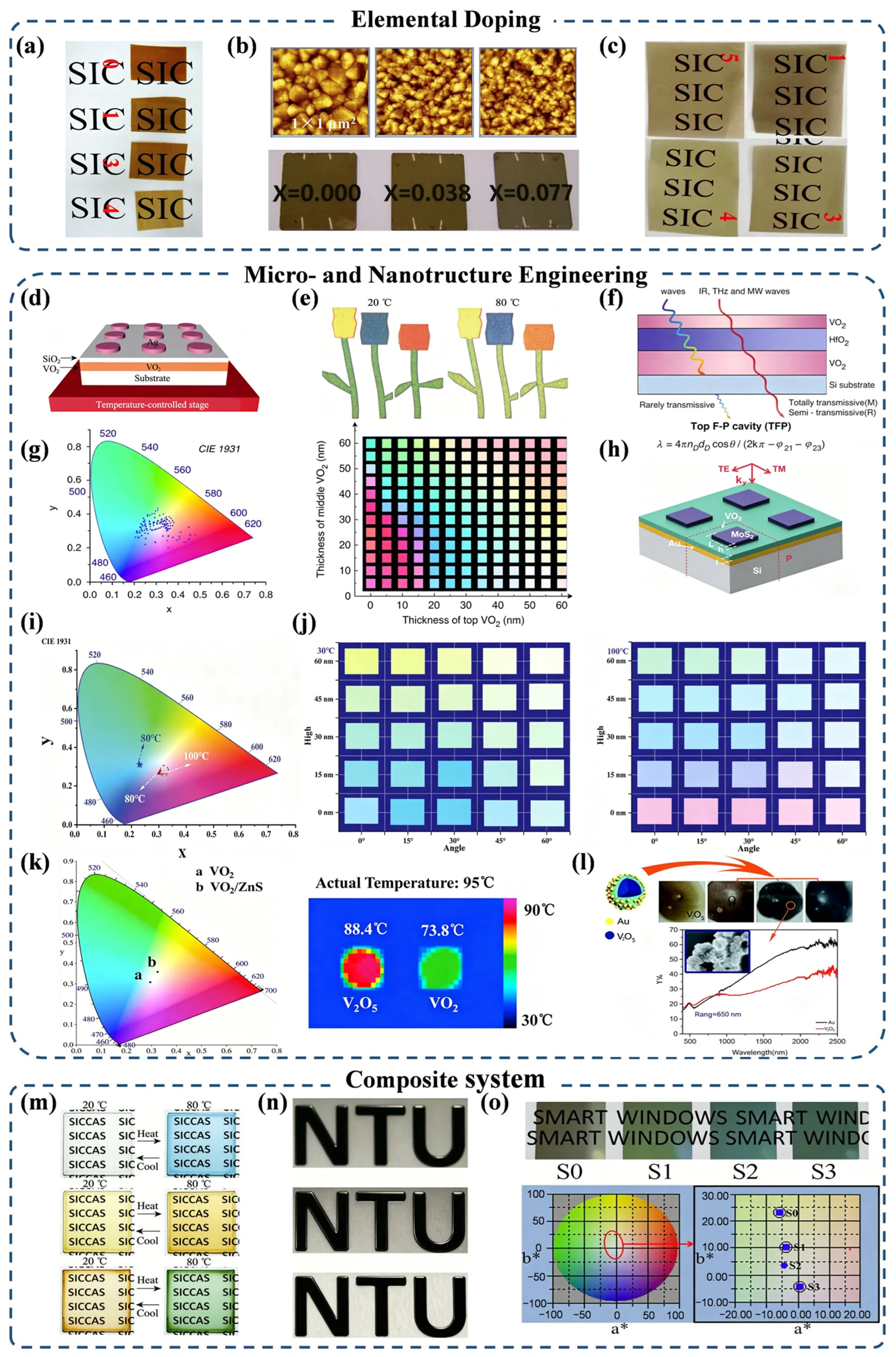
Color regulation of VO_2_. (**a**) Photographs of VO_2_ films with different Mg doping levels: Samples 0, 1, 3, and 4 correspond to Mg doping levels of 0.0, 2.1, 3.8, and 7.0 at%, respectively [76], copyright 2013, Royal Society of Chemistry. (**b**) AFM images of V_1−x_Zn_x_O_2_ films with different Zn doping concentrations: samples correspond to Zn doping levels of 0.0, 3.8, and 7.7 at% (**top**) and their corresponding macroscopic images (**bottom**) [77], copyright 2014, Elsevier. (**c**) Macroscopic images of Zr-doped and W-Zr co-doped VO_2_ films [78], copyright 2014, Royal Society of Chemistry. The numbers 1, 3, 4 and 5 in the picture represent different samples. (**d**) VO_2_ film with a designed periodic Ag nanoarray and (**e**) a schematic of its color change in response to temperature [81], copyright 2018, Wiley VCH. (**f**) Design of a VO_2_-based cascaded TFP structure and (**g**) color regulation results for different VO_2_ thickness combinations: the left panel shows CIE 1931 chromaticity coordinates, and the right panel shows a thickness-color mapping [82], copyright 2024, Springer Nature. (**h**) Schematic diagram of the MoS_2_/VO_2_/Au multilayer metasurface, (**i**) distribution of CIE 1931 chromaticity coordinates at different temperatures, and (**j**) dynamic structural color under high- and low-temperature conditions for various MoS_2_ layer thicknesses and incident angles [83], copyright 2023, De Gruyter. (**k**) Comparison of CIE 1931 chromaticity coordinates between VO_2_ and VO_2_/ZnS core–shell structures (**left**) and infrared thermal imaging results of VO_2_ and V_2_O_5_ under high-temperature conditions (**right**) [84], copyright 2018, Elsevier. (**l**) Characterization of the structure, color evolution, and optical performance of VO_2_@SiO_2_@Au core-shell nanoparticles [85], copyright 2016, Royal Society of Chemistry. (**m**) Temperature-responsive color changes in composite films of VO_2_ and IL-Ni-Cl complexes [87], copyright 2016, American Chemical Society. (**n**) Photograph showing changes in the clarity of NTU patterns [88], copyright 2016, Royal Society of Chemistry. (**o**) Changes in color and clarity of smart windows in different composite systems [89], copyright 2015, Royal Society of Chemistry. a* and b* represent the chromaticity coordinates in the CIE.

Elemental doping can also lighten or alter the color by modulating the band structure and reducing visible light absorption. Early research focused on single-component metal doping, utilizing changes in the optical bandgap to optimize color. Zhou et al. [76] pioneered research on Mg-doped modification. They prepared Mg-doped VO_2_ nanoparticles via the hydrothermal method. At 3.8 at% Mg doping, the absorption edge of the film shifted blueward from 490 nm to 440 nm, and the optical bandgap widened to 2.4 eV, resulting in increased brightness and a significant lightening of the yellow hue. The corresponding physical results are shown in Figure 5a. Jiang et al. [77] extended their research to Zn-doped systems. V_1−x_Zn_x_O_2_ films prepared via magnetron sputtering (see the upper half of Figure 5b) exhibited a markedly reduced yellow tint (see the lower half of Figure 5b) compared to pure VO_2_ films of the same thickness, and their luminous transmittance was significantly improved. Shen et al. [78] further investigated the behavior of Zr doping. While undoped VO_2_ exhibits an optical bandgap of 1.59 eV, the bandgap widened to 1.89 eV at a Zr doping concentration of 9.8 at%. Consequently, the yellow tint of the film was significantly reduced, and the luminous transmittance reached 60.4%. Unlike the aforementioned metal doping strategies that primarily focus on hue optimization, W doping offers additional capabilities for color expression and demonstrates unique advantages in color regulation. Christopher et al. [79] prepared W-doped VO_2_ films using atmospheric pressure chemical vapor deposition. They found that the film color changes progressively with increasing doping concentration, transitioning from an initial yellow/brown to green/blue. This approach not only lowers the phase transition temperature and optimizes optical performance but also opens new avenues for the color regulation of VO_2_. Building on this, co-doping systems have emerged as a key development direction to simultaneously achieve multiple performance enhancements, including phase transition temperature regulation and color optimization. Building on their research on Zr doping, Shen et al. [78] further synthesized W-Zr co-doped VO_2_ nanoparticles. While significantly lowering T_c_ to 28.6 °C, they maintained good optical performance and achieved color optimization, thereby realizing the simultaneous optimization of temperature regulation, color regulation, and optical performance. A photograph of the resulting film is shown in Figure 5c.

### 4.2. Micro- and Nanostructure Engineering

Micro- and nanostructure engineering is another important method for improving the intrinsic color of VO_2_. By constructing micro- and nanostructures such as plasmonic arrays, resonant cavities, metasurfaces, and core–shell structures, it is possible to overcome the limitations of the material’s intrinsic absorption and further optimize the film color. In a plasmonic array-based system, Zhou et al. [80] utilized self-assembled hexagonal close-packed polystyrene (PPAs) templates to embed periodic arrays of Ag particles into VO_2_ (M1) films. The study demonstrated that by adjusting the specific surface area of Ag-PPAs, strong light absorption in the 630–730 nm wavelength range (brownish-yellow) could be achieved, thereby diluting the brownish-yellow hue of the VO_2_ film while simultaneously lowering T_c_ to 57.5 °C. Building on this, Shu et al. [81] further designed Ag nanoarrays on the surface of VO_2_ films using ion beam etching (see Figure 5d), achieving strong visible light absorption via the Ag LSPR effect. By varying the array period and the diameter of the nanodisks, the visible light hue can be artificially tailored. Furthermore, leveraging the MST properties of VO_2_, dynamic color switching under temperature regulation was achieved, as shown in Figure 5e. Fabry-Pérot resonators are also a key approach for regulating structural color. Wei et al. [82] designed a cascaded VO_2_-based Fabry-Pérot resonator (see Figure 5f), using the cavity layer thickness to alter the resonance wavelength shift before and after the phase transition. The spectral shift in the visible light region reached 60 nm, supporting continuous color changes and expanding the wavelength range for structural color regulation. The color variation trends for different VO_2_ thickness combinations and the corresponding CIE 1931 chromaticity coordinate distributions are shown in Figure 5g. Furthermore, the development of metasurface structures has further enriched the strategies for dynamic structural color regulation. The MoS_2_/VO_2_/Au multilayer metasurface designed by Cheng et al. [83] (see Figure 5h) utilizes the change in refractive index before and after the VO_2_ phase transition to modulate the Fabry-Pérot resonance, achieving dynamic switching of structural color in the visible light region. Specifically, the film exhibits a lime-green color at room temperature and transitions to a yellowish-brown hue after the phase transition. It demonstrates high color response sensitivity, and the color range can be customized by adjusting the MoS_2_ layer thickness and the incident angle, offering a new solution for smart window design. The temperature-responsive structural color characteristics of this multilayer metasurface are shown in Figure 5i,j. Core–shell structures also demonstrate excellent potential for color regulation. Ji et al. [84] synthesized VO_2_/ZnS core–shell nanoparticles via a uniform precipitation method. The ZnS shell not only transformed the VO_2_ particles from blue-black to a gray-green hue more suitable for visible light camouflage scenarios but also significantly enhanced their oxidation resistance. The chromaticity coordinates and infrared thermal imaging results at high temperatures are shown in Figure 5k. Building on this, researchers further explored the tuning effects of multicomponent core–shell systems. Lu et al. [85] prepared VO_2_@SiO_2_@Au core–shell particles using a solution method. By varying the size of the Au particles, they induced a series of color changes in the composite film ranging from brick red to purple and blue, while simultaneously lowering the T_c_ by approximately 6 °C and improving stability. The structure, color evolution, and optical performance characterization of these core–shell nanoparticles are shown in Figure 5l.

### 4.3. Composite Systems

Compositing VO_2_ with other materials has proven to be an effective method for color optimization. Zhu et al. [86] prepared films by compositing VO_2_ nanoparticles with a Co^II^-Br-TMP complex. Upon heating, the films exhibited a significant color change from brownish-yellow to dark green, along with excellent stability and optical performance. Furthermore, the team conducted further research on IL-Ni-Cl (ionic liquid-nickel-chlorine) composites [87], finding that while overcoming the volatility of organic components, these composites enabled the film to change distinctly from light brown to dark green as the temperature increased. The solar modulation efficiency and luminous transmittance reached as high as 26.45% and 66.44%, respectively. The color-changing effect of the film is shown in Figure 5m. In addition to organic composite systems, polymer hydrogels have also been employed for the composite modification of VO_2_. Zhou et al. [88] combined VO_2_ with PNIPAm hydrogel material. As shown in Figure 5n, this composite film appears transparent at low temperatures but becomes a translucent brown at high temperatures. While achieving this change in clarity, the system also exhibits a solar modulation efficiency of 34.7% and a luminous transmittance of 62.6%, providing a novel design approach for the development of smart window materials that combine visual comfort with energy-saving benefits. Multilayer composite structures can also achieve color regulation. Hu et al. [89] prepared a VO_2_/Au/VO_2_ sandwich structure using magnetron sputtering and rapid-thermal annealing processes. By leveraging the LSPR effect to cause a red shift in the resonance peak, the film’s appearance changed from brownish-yellow to blue-green (see Figure 5o), while this structure also reduced T_c_ to 42.5 °C.

## 5. Optical Performance Regulation

The optical performance of VO_2_-based smart windows is typically characterized by two key parameters: visible light transmittance (T_lum_) and solar modulation efficiency (ΔT_sol_). Unless otherwise noted, all T_lum_ values cited throughout this manuscript refer to the luminous transmittance measured under the low-temperature insulating cold-state, following the common-used standard in this research community. Synergistically enhancing both parameters has been a key research focus in this field. In recent years, researchers have primarily optimized the optical performance of VO_2_ through methods such as micro- and nanostructure design, multilayer film design, and inorganic–organic composite modification. A summary of the progress in optical performance regulation of VO_2_-based smart windows is summarized in Table 3.

### 5.1. Micro- and Nanostructure Design

Controlling micro- and nanostructural characteristics, such as porosity, morphology, and periodic arrangement of the film, enables the optimization of optical constants and light scattering behavior without altering the chemical composition of VO_2_. This approach therefore represents an important method for simultaneously enhancing visible light transmittance and solar modulation efficiency.

**Figure 6:**
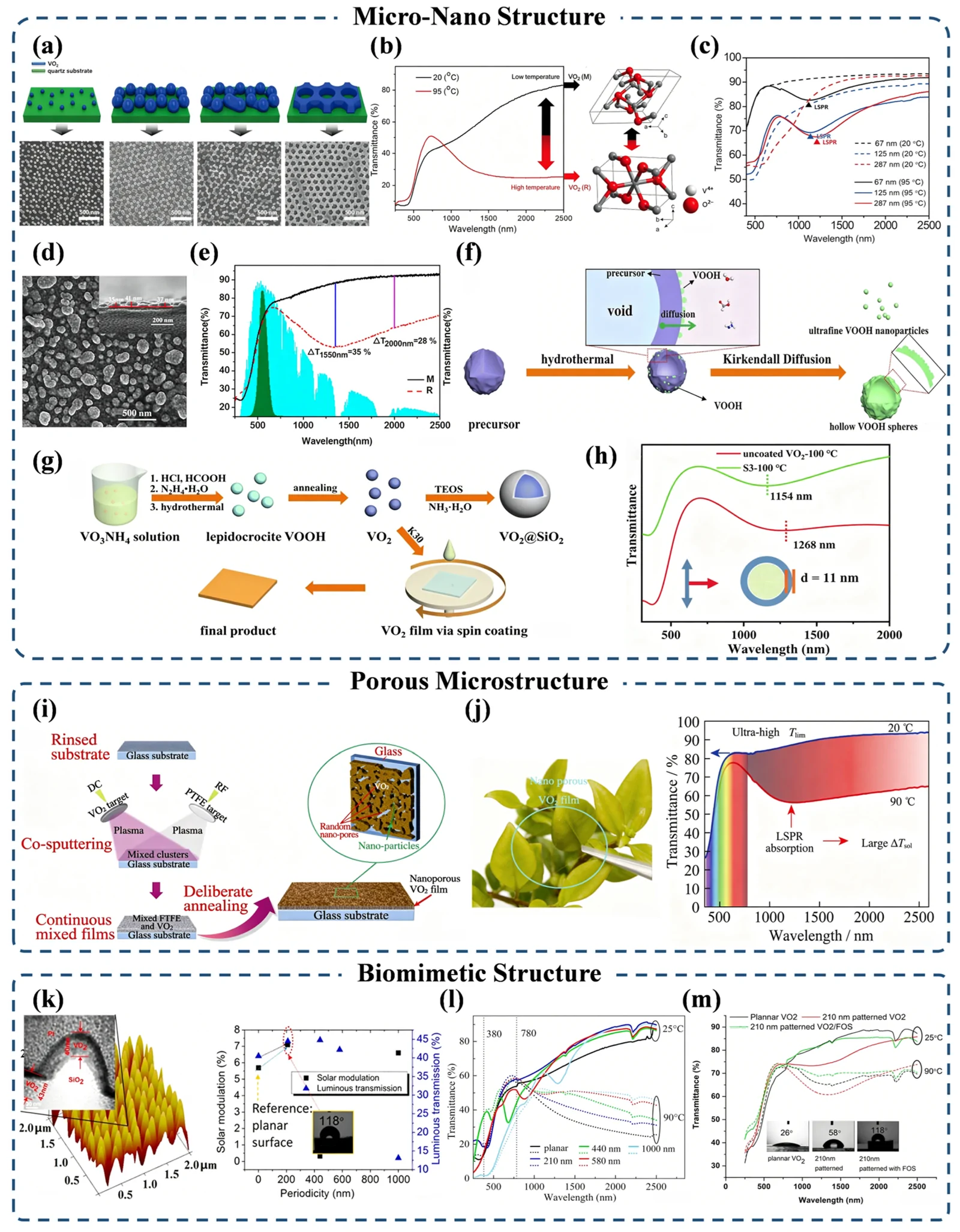
Design of VO_2_ micro- and nanostructure: (**a**) Preparation process of two-dimensional ordered nanopatterned VO_2_ films. (**b**,**c**) Transmission spectra of VO_2_ films with different nanopattern sizes [90], copyright 2017, American Chemical Society. (**d**) SEM image of a double-sided VO_2_ micro- and nanostructured film and (**e**) transmission spectra of this structure [91], copyright 2020, American Chemical Society. (**f**) Schematic of the preparation of ultrafine VOOH and (**g**) SiO_2_-coated structures, as well as (**h**) transmission spectra of SiO_2_-coated VO_2_ nanoparticle films [92], copyright 2024, Royal Society of Chemistry. (**i**) Preparation process of a nanoporous VO_2_ film, (**j**) photograph, and transmission spectra [57], copyright 2019, American Chemical Society. The blue curve represents the transmittance at 20 °C, the red curve represents the transmittance at 90 °C, and the vertical rainbow-colored band indicates the visible-light wavelength range. (**k**) Surface morphology of a moth-eye-inspired VO_2_ film and (**l**,**m**) optical performance [95], copyright 2014, American Chemical Society. The solid lines represent the transmittance spectra at 25 °C, and the dashed lines represent the transmittance spectra at 90 °C.

Ordered micro- and nanoarrays are one of the mainstream approaches in micro- and nanostructure design. Ke et al. [90] prepared a series of two-dimensionally ordered VO_2_ nanostructures using nanospherical lithography. The resulting nanopatterns, shown in Figure 6a, endow the film with a significant LSPR effect, effectively improving its optical performance and achieving a synergistic enhancement of both properties (T_lum_ = 46%, ΔT_sol_ = 13.2%). The corresponding transmission spectra are shown in Figure 6b,c. To simplify the fabrication process, Dou et al. [91] developed a simple and versatile inorganic sol–gel method (see Figure 6d), uniformly dispersing VO_2_ nanoparticles on both sides of a fused silica substrate to excite a double-sided LSPR effect. By tuning the sol–gel and heat treatment conditions, both the nanoparticle morphology and the LSPR response can be precisely controlled, yielding a synergistic enhancement of T_lum_ to 68.2% and ΔT_sol_ to 11.7%. The corresponding transmission spectra are presented in Figure 6e. Notably, after 1500 thermal cycles, the performance degradation of this double-sided structure did not exceed 1%, demonstrating excellent stability. In addition, Wu et al. [92] exploited the Kirkendall effect to synthesize ultrafine VOOH nanoparticles hydrothermally, which were then annealed and converted into morphologically stable VO_2_(M) nanoparticles (Figure 6f). The resulting film achieved a T_lum_ of 56.45% and a ΔT_sol_ of 14.95%. To further enhance performance, a SiO_2_ shell was introduced as a protective coating (Figure 6g). After modification, T_lum_ rose to 62.29% while ΔT_sol_ was maintained, and the stability was markedly improved. The particle microstructure and corresponding transmission spectra are displayed in Figure 6h. Beyond conventional vertical nanoarray configurations, oblique-angle deposition enables the construction of anisotropic tilted columnar VO_2_ architectures. Bhupathi et al. [93] utilized this technique to fabricate sculptured columnar thermochromic coatings. The oriented inclined microstructure yields unique angle-dependent thermochromic characteristics, delivering a T_lum_ of 43% and ΔT_sol_ of 11%. This design effectively adapts to dynamic solar incident angles, greatly improving the environmental adaptability of VO_2_ nanoarray-based smart windows.

Porous micro- and nanostructures offer another effective route for enhancing optical performance. Kang et al. [94] fabricated nanoporous VO_2_ films via a polymer-assisted deposition method. The porous architecture effectively lowered the optical constants in the visible region, yielding a T_lum_ of 43.6% and a ΔT_sol_ of 14.1%. Optical simulations further suggested that increasing the porosity could lead to additional performance gains. Long et al. [57] further refined the porous architecture. The optimized nanoporous VO_2_ film delivered T_lum_ values of 78.0% and 72.8% in the insulating and metallic states, respectively, while maintaining a ΔT_sol_ of 14.1%. The fabrication process and simulated transmission spectra are presented in Figure 6i,j. Moreover, the unique microstructure enables multi-angle anti-reflection in the visible range, highlighting its potential for intelligent solar regulation in vehicle windshields.

Bio-inspired architectures, including moth-eye, offer an alternative pathway to enhance optical performance without modifying the chemical composition of VO_2_. Qian et al. [95] fabricated a moth-eye-inspired VO_2_ nanoarray film. The conical nanostructures create a gradient refractive index profile that suppresses interfacial reflection, yielding simultaneous enhancements in T_lum_ and ΔT_sol_. Compared with a planar film, the 210 nm-period moth-eye structure increased T_lum_ by ~10% and ΔT_sol_ by ~24.5%. In addition, the nanostructured surface yielded a contact angle of ~118°, imparting hydrophobic self-cleaning functionality. The corresponding surface morphology and transmission spectra are displayed in Figure 6k–m. In contrast to the moth-eye design, which relies on a graded refractive index at the interface, photonic crystals exploit the photonic bandgap effect to realize dynamic spectral regulation. Apart from fixed-shape nanostructure arrays, particle-size manipulation serves as another promising micro-nanostructure optimization strategy. Liang et al. [96] adopted bimodal-sized VO_2_ nanoparticles (≈50 nm and ≈100 nm) for particle-size engineering inside a Fabry-Pérot cavity device. Benefiting from the synergistic effect of dual-dimension nanoparticles, the optimized sample achieves T_lum_ = 56.09% and ΔT_sol_ = 16.72%. The bimodal-size particles balance near-infrared attenuation at high temperatures and favorable visible light transmission at low temperatures, easing the trade-off between T_lum_ and ΔT_sol_. Femtosecond laser surface patterning serves as a feasible post-processing technique for fabricating periodic VO_2_ micro-nanostructures. In the work of Bhupathi et al. [97], one-dimensional VO_2_ nanograting arrays were fabricated by femtosecond laser etching. Raised grating height and reduced grating periodicity contribute to the promotion of polarization-sensitive T_lum_. The processed VO_2_ thin-film delivers improved near-infrared transmittance and solar modulation performance, and its maximum ΔT_sol_ reaches 10.9%.

### 5.2. Multilayer Film Design

Multilayer film design is another important method for improving the optical performance of VO_2_-based smart windows. Jin et al. [98] conducted early systematic research on a TiO_2_ (25 nm)/VO_2_ (50 nm)/TiO_2_ (25 nm) sandwich anti-reflective structure. They found that this structure increased T_lum_ from 30.9% to 57.6%—an 86% increase—while the phase transition temperature decreased slightly to 57.5 °C. Building on this, Long et al. [71] further developed a WO_3_/VO_2_/WO_3_ sandwich structure, increasing the film’s T_lum_ from 37.2% to 55.4% while lowering the phase transition temperature to 52 °C. Notably, the hysteresis loop became steeper near the transition temperature, an effect attributed to the modulation of interfacial stress and diffusion. Moreover, the stability of this multilayer film under high-temperature and high-humidity conditions was nearly four times that of a single-layer VO_2_ film. Additionally, Xu et al. [99] conducted optical optimization studies on anti-reflective (AR) coatings for VO_2_-based smart windows. Theoretical calculations showed that for a single-layer VO_2_ film with a refractive index n ≈ 2.2 and a thickness of 50 nm, T_lum_ could be increased from 32% (uncoated) to 55%. This study also identified suitable anti-reflective layer materials, including TiO_2_, HfO_2_, ZrO_2_, ZnO, and ZnS, providing key references for the design of multilayer film structures. Among these candidate dielectrics, HfO_2_ stands out for its comprehensive performance. Chang et al. [100] fabricated VO_2_/HfO_2_ bilayer films, in which HfO_2_ functions as both an anti-reflection coating and a hydrophobic barrier. Owing to its proper refractive index, the bilayer achieves improved T_lum_ while preserving good ΔT_sol_. Meanwhile, the HfO_2_ layer suppresses moisture-triggered degradation of VO_2_, and accelerated high-temperature–high-humidity tests verify its significantly extended service lifetime. Furthermore, Sol et al. [101] proposed a unique strategy of temperature-dependent optical impedance matching to approach the intrinsic optical performance limit of VO_2_ multilayer films. In their experiment, excellent thermochromic properties with T_lum_ = 48.9% and ΔT_sol_ = 21.8% were achieved, which provides important guidance for the design of high-performance VO_2_ multilayer-film smart windows.

**Figure 7:**
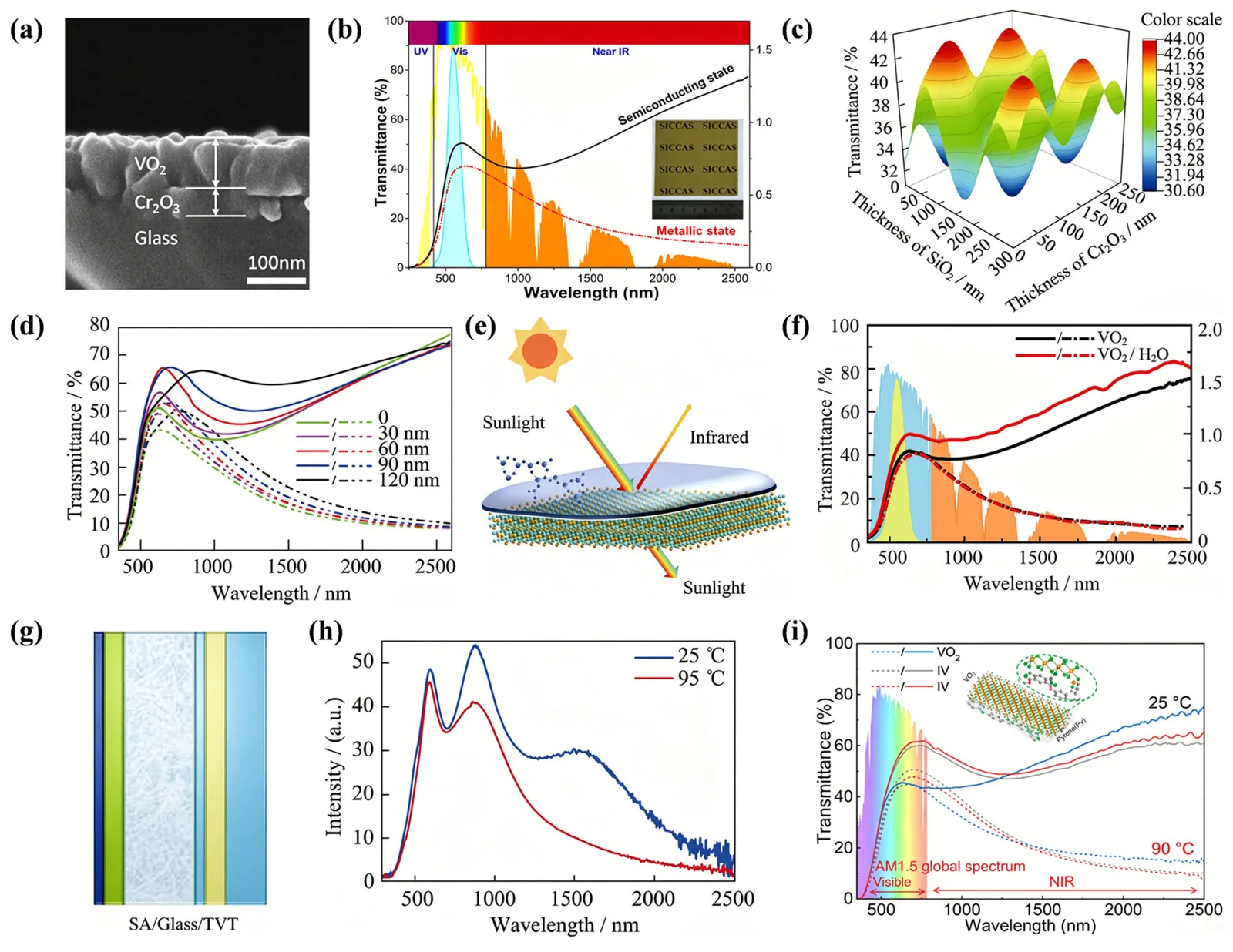
Design of multilayer film structures: (**a**) Cross-sectional SEM image of a Cr_2_O_3_/VO_2_ bilayer film, along with (**b**) its photograph and transmission spectrum [58], copyright 2017, American Chemical Society; (**c**) 3D surface plot of the optical performance of a Cr_2_O_3_/VO_2_/SiO_2_ multilayer film as a function of Cr_2_O_3_ and SiO_2_ thickness. (**d**) Transmission spectra (350–2600 nm) of CVS structures with different SiO_2_ thicknesses at 25 °C (solid line) and 90 °C (dashed line) [102], copyright 2018, Elsevier. (**e**) Schematic diagram illustrating the working principle of the novel VO_2_/H_2_O smart anti-reflection structure; (**f**) comparison of transmission spectra for VO_2_ and VO_2_/H_2_O thin films [103], copyright 2019, American Chemical Society. (**g**) Schematic diagram of the SA/Glass/TVT multilayer film structure; (**h**) transmission spectra of the TVT structure at different temperatures [105], copyright 2017, Elsevier. (**i**) Transmission spectra and schematic diagram of the ITO/VO_2_/ITO flexible thin film [106], copyright 2022, American Chemical Society. The yellow, blue and orange shaded regions represent the visible, near-infrared and mid-infrared bands, respectively; the solid lines denote the spectra at 25 °C, and the dashed lines denote the spectra at 90 °C.

The introduction of buffer layers and composite anti-reflective structures has further expanded the functional boundaries of multilayer films. Chang et al. [58] used Cr_2_O_3_ as a template to prepare a Cr_2_O_3_/VO_2_ bilayer film via magnetron sputtering. An SEM image of the bilayer is presented in Figure 7a. Relative to a single-layer VO_2_ film, the bilayer exhibited substantially enhanced solar modulation, achieving synergistic T_lum_ and ΔT_sol_ values of 46.0% and 12.2%, respectively. The corresponding transmission spectrum is shown in Figure 7b. Subsequently, the same team [102] continued to optimize the structure, designing a Cr_2_O_3_/VO_2_/SiO_2_ (CVS) multilayer energy-saving window. By using Cr_2_O_3_ and SiO_2_ as anti-reflective layers, they increased the film’s T_lum_ to 54.0% and ΔT_sol_ to 16.1%, approaching the theoretical limit (~17%) calculated for VO_2_-based multilayer films. The corresponding transmission spectra are shown in Figure 7c,d. Building simulation tests indicate that this CVS multilayer energy-saving window can significantly reduce indoor temperatures in summer and decrease air conditioning energy consumption, contributing to the achievement of development goals for building carbon reduction and energy efficiency improvement. In addition to traditional inorganic dielectric layers, various novel functional anti-reflective systems have also attracted attention. Xu et al. [103] proposed a novel VO_2_/H_2_O smart anti-reflective structure. This approach utilizes a removable solvent layer to create a “removable” AR coating, which significantly enhances the film’s optical performance while offering the advantages of low cost, energy efficiency, and environmental friendliness. Compared to traditional AR layers, it is easier to fabricate and commercialize; its structure and transmission spectra are shown in Figure 7e,f.

In addition to optimizing optical performance, multilayer structures can also endow smart windows with multifunctional properties. Zheng et al. [104] improved upon the traditional TiO_2_/VO_2_/TiO_2_ (TVT) structure by fabricating a Glass/TiO_2_(R)/VO_2_(M)/TiO_2_(A) multilayer structure, which effectively enhanced the film’s optical performance. In this structure, the bottom TiO_2_(R) layer enhances the crystallinity of VO_2_, while the top TiO_2_(A) layer confers superhydrophilicity and photocatalytic properties to the film, enabling the smart window to simultaneously provide energy-saving, anti-fog, and self-cleaning functions. Additionally, Sun et al. [105] combined the TVT structure with a SiO_2_/AZO mid-infrared reflective film to obtain an SA/Glass/TVT multilayer film structure, whose structure and transmission spectra are shown in Figure 7g,h. This film achieves low transmittance in the 1350–2500 nm wavelength range, effectively improving skin comfort. Furthermore, Cao et al. [106] fabricated a flexible ITO/VO_2_/ITO (IVI) film on a colorless polyimide substrate, enabling direct attachment to glass surfaces. The device model and transmission spectrum are displayed in Figure 7i. This flexible system combines high optical performance with robust stability, opening new opportunities for wearable devices and curved architectural facades.

### 5.3. Inorganic–Organic Composite Modification

**Figure 8:**
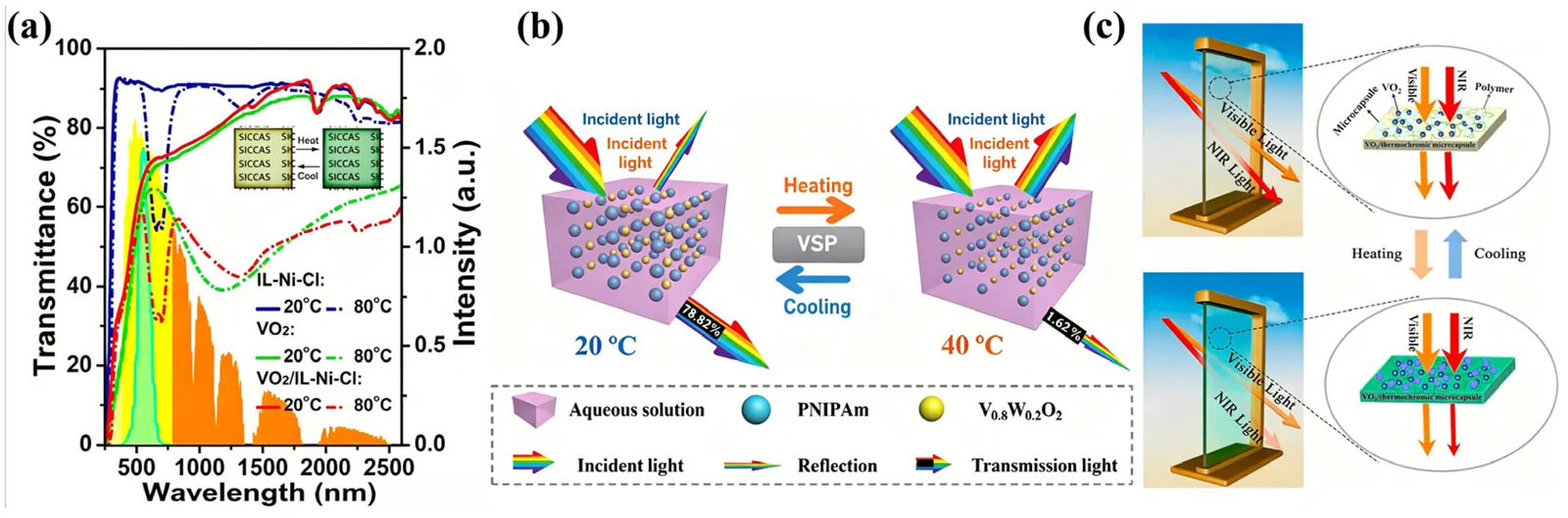
Inorganic–organic composite modification: (**a**) Transmission spectra of VO_2_/Ni-Cl-IL composite films and photographs showing color changes at 20 °C and 80 °C [87], copyright 2016, Royal Society of Chemistry. The blue, green and red curves represent VO_2_, Ni-Cl-IL and VO_2_/Ni-Cl-IL samples, respectively; the solid lines denote the spectra at 20 °C, and the dashed lines denote the spectra at 80 °C. The yellow, green and orange shaded regions correspond to the visible, near-infrared and mid-infrared wavelength bands. (**b**) Schematic diagram of the operation of a VSP thermochromic microgel system based on V_1−x_W_x_O_2_@SiO_2_ and PNIPAm hydrogels [109], copyright 2021, Royal Society of Chemistry. (**c**) Schematic diagram of the structure and operating principle of a VO_2_ composite film capable of switching between cool and warm tones [110], copyright 2023, Elsevier.

In recent years, researchers have proposed inorganic–organic composite modification as a method to enhance the optical performance of VO_2_-based smart windows. Zhou et al. [107] explored the implementation of poly(N-isopropylacrylamide) hydrogel thin-film in thermochromic smart window systems. The hydrogel film with optimized-thickness attained a favorable comprehensive thermochromic performance, achieving an average T_lum_ of 70.7% and ΔT_sol_ of 25.5%, alongside a reduced phase-transition temperature of around 32 °C. Among these, ionic liquid composite systems have been the most extensively investigated. Zhu et al. [87] combined a metal ion ligand conversion system that absorbs at specific wavelengths with VO_2_ nanopowder, successfully producing VO_2_/Ni-Cl-IL composite films. Compared to pure VO_2_ films, the optical performance of these composite films was significantly improved (T_lum_ = 55.19%, ΔT_sol_ = 26.45%), and the color also underwent a significant change, shifting from a light brown at low temperatures to a deep green at high temperatures. The transmission spectra and color changes are shown in Figure 8a. Building on this, Chen et al. [108] further investigated the composite of VO_2_ nanoparticles with a nickel-bromine ionic liquid (Ni–Br–IL). The resulting VO_2_/Ni–Br–IL composite film also exhibited excellent optical performance, with T_lum_ values as high as 65.9% at low temperature and 55.3% at high temperature, and ΔT_sol_ increased to 16.1%. Furthermore, combining doped VO_2_ with thermosensitive hydrogels can further overcome the performance limitations of traditional systems. Zhang et al. [109] designed a novel VSP thermochromic microgel system centered on a tungsten-doped vanadium dioxide core–shell structure (V_1−x_W_x_O_2_@SiO_2_) and a thermosensitive PNIPAm hydrogel material, as shown in Figure 8b. This system reduces T_c_ to approximately 30 °C through W doping, while utilizing SiO_2_ doping to regulate the structural changes of the PNIPAm microgel, thereby achieving synergistic regulation in both the visible and near-infrared wavelength bands. Ultimately, this results in a T_lum_ of up to 92.48% and a ΔT_sol_ of up to 77.20% for the film, significantly broadening the spectral regulation range of VO_2_-based smart windows. In addition to hydrogels, thermochromic microcapsules are also commonly used in the design of inorganic–organic composite systems. Ji et al. [110] dispersed VO_2_ nanoparticles, synthesized via a one-step hydrothermal route, together with thermochromic microcapsules in an acrylic polyol resin matrix. The resulting composite film can switch between cool and warm tones, and its structure is illustrated in the right half of Figure 8c. Excellent optical performance is realized through the synergistic action of the microcapsules (visible-range modulation) and VO_2_ (near-infrared modulation), yielding a T_lum_ of 48.70% and a ΔT_sol_ of 18.92% at low temperatures. Concurrently, the microcapsules undergo a colorless-to-green transition above 40 °C (left half of Figure 8c), imparting pronounced thermochromic color-shifting behavior. This dual-responsive design offers a compelling strategy for smart windows that integrate energy efficiency with visual comfort. It should be noted that composite strategies producing obvious visible color variation upon phase transition have inherent practical drawbacks for real-world smart window applications. End-users generally prefer color-neutral glazing materials. Furthermore, spatial temperature inhomogeneity across the window surface may lead to undesired patchy and mottled visual appearances under non-uniform thermal conditions.

Against the above-mentioned limitations of color-switching composite systems, embedding VO_2_-based nanoparticles into transparent inorganic or organic coating-matrix materials emerges as a highly practical modification direction. This composite-construction strategy can optimize the thermochromic optical property while retaining the inherent color-neutral appearance of coatings and avoiding unwanted thermally triggered color drift. Zhu et al. [111] adopted an annealing-assisted method to synthesize high-crystallinity V_x_W_1−x_O_2_@SiO_2_ core–shell nanoparticles for thermochromic smart window coatings. The post-annealing process reduced internal defects and boosted nanoparticle crystallinity. The optimized composite coating realized balanced thermochromic properties, with a T_lum_ of 52.2% and ΔT_sol_ of 17.3%. Its critical phase-transition temperature was adjusted down to 25.2 °C, satisfying the practical deployment demand of architectural windows. Targeting seasonal-changing environments, Mann and co-workers [112] optimized VO_2_-based composite thermochromic coatings for climate-adaptive usage. The optimized coating delivered a T_lum_ of 70.0% and ΔT_sol_ of 20.1%. Outdoor field tests verified its stable optical property under variable seasonal conditions, and building-energy simulation validated its energy-saving capability for residential buildings. Building upon their outdoor environmental experiments, Mann’s group [113] further investigated VO_2_-SiO_2_ composite thermochromic coatings installed within insulated glazing units. The optimal-performing coating delivers a T_lum_ of 67.1% and a ΔT_sol_ of 22.9%, which reaches 59% of the calculated theoretical upper-limit. A set of feasible optimization strategies was also proposed to bridge the gap between practical measured performance and the theoretical performance ceiling. Apart from foregoing experiments on VO_2_-SiO_2_ coatings, Schläfer et al. [114] explored the theoretical performance limit of VO_2_-based composite-coating. They analyzed how nanoparticle filling fraction and film thickness affect thermochromic properties, which supplies effective references for subsequent coating optimization.

Besides polymer-matrix composite systems, liquid-crystal materials act as outstanding organic substrates for VO_2_-integrated hybrid smart window devices. Barinova’s group has completed a sequential series of studies on composite systems consisting of nanoporous-microparticle-doped liquid crystal and functional VO_2_ thin-film. Their preliminary work in 2021 fabricated a dual-layer non-reciprocal scattering metamaterial smart window. The solar-light modulation performance reached the optimum when the VO_2_ thin-film faced outward, and the device delivered millisecond-level electro-optical response speed [115]. Subsequently in 2023, the group embedded nanoporous VO_2_ microparticles into the liquid-crystal matrix. The metamaterial composite switched between scattering and transparent states under applied voltage, and the micro-sized VO_2_ microparticles delivered favorable infrared heat-blocking capacity [116]. In 2024, the team introduced an ultrafast pulsed-laser-patterned nanostructured VO_2_ metasurface. The laser-etched nanograting lines guided the alignment of liquid-crystal molecules and further boosted the thermochromic property of the integrated device [117]. Another 2024 investigation adopted oblique-angle-deposited nanostructured VO_2_ thin-film paired with tunable-scattering NMP-liquid-crystal medium. Owing to its asymmetric layered configuration, the device exhibited non-reciprocal optical response. The optimized sample obtained enhanced infrared-shielding capability and sharply reduced operating voltage for visible light transmittance tuning, laying a foundation for low-power-consumption smart window elements [118].

**Table 3:** Optical performance regulation in VO_2_-based smart windows.

Authors	Structure	T_lum_	ΔT_sol_	Descriptions	Ref.
Long et al.	Micro- and nanostructure	78.0% (low temperature) 72.8% (high temperature)	14.1%	Porous micro- and nanostructured VO_2_ films	[57]
Ke et al.	Micro- and nanostructure	46%	13.2%	Hexagonal patterned VO_2_ nanoparticle array	[90]
Dou et al.	Micro- and nanostructure	68.2%	11.7%	Double-sided VO_2_ nanoparticle coating	[91]
Wu et al.	Micro- and nanostructure	62.29%	14.95%	Goethite-type VOOH ultrafine nanoparticles with SiO_2_ shells	[92]
Kang et al.	Micro- and nanostructure	43.6%	14.1%	Porous micro- and nanostructured VO_2_ film	[94]
Liang et al.	Micro- and nanostructure	56.09%	16.72%	50 nm and 100 nm dual-dimensional VO_2_ nanoparticles are assembled inside a Fabry-Pérot cavity	[96]
Bhupathi et al.	Micro- and nanostructure	43%	11%	Oblique-angle-deposited sculptured tilted-column VO_2_ coatings	[97]
Chang et al.	Multilayer film structure	46.0%	12.2%	Cr_2_O_3_/VO_2_ bilayer film structure	[58]
Long et al.	Multilayer film structure	55.4%	/	WO_3_/VO_2_/WO_3_ sandwich anti-reflection structure	[71]
Jin et al.	Multilayer film structure	57.6%	/	TiO_2_/VO_2_/TiO_2_ sandwich anti-reflection structure	[98]
Xu et al.	Multilayer film structure	42.5%	18.2%	VO_2_/H_2_O bilayer film structure	[99]
Chang et al.	Multilayer film structure	/	/	VO_2_/HfO_2_ bilayer films	[100]
Sol et al.	Multilayer film structure	48.9%	21.8%	Proposed temperature-dependent optical impedance-matching strategy	[101]
Chang et al.	Multilayer film structure	54.0%	16.1%	Cr_2_O_3_/VO_2_/SiO_2_ (CVS) trilayer film structure	[102]
Cao et al.	Multilayer film structure	46.5%	15.65%	Flexible ITO/VO_2_/ITO trilayer film structure	[106]
Zhou et al.	Inorganic–organic composite modification	70.7%	25.5%	Poly(N-isopropylacrylamide) thermo-responsive hydrogel thin-film with reduced T_c_	[107]
Chen et al.	Inorganic–organic composite modification	65.9% (low temperature) 55.3% (high temperature)	16.1%	VO_2_/Ni-Br-IL composite film	[108]
Zhang et al.	Inorganic–organic composite modification	92.48%	77.20%	Composite of tungsten-doped vanadium dioxide core–shell structures (V_1−x_W_x_O_2_@SiO_2_) and PNIPAm hydrogel material	[109]
Ji et al.	Inorganic–organic composite modification	48.70%	18.92%	Composite of VO_2_ nanoparticles and thermochromic microcapsules with acrylic polyol resin	[110]
Zhu et al.	Inorganic–organic composite modification	52.2%	17.3%	Annealing-assisted high-crystallinity V_x_W_1−x_O_2_@SiO_2_ core–shell nanoparticle coating, T_c_ reduced to 25.2 °C	[111]
Mann et al.	Inorganic–organic composite modification	70.0%	20.1%	Climate-adaptive VO_2_ composite thermochromic coating validated by outdoor seasonal-condition tests	[112]
Mann et al.	Inorganic–organic composite modification	67.1%	22.9%	VO_2_-SiO_2_ composite coating applied in insulated glazing units, achieving 59% of the theoretical performance limit	[113]
Madhuri et al.	Inorganic–organic composite modification	/	/	Non-reciprocal light-scattering hybrid VO_2_ and liquid-crystal composite film for visible-infrared radiation dynamic regulation	[115]
Barinova et al.	Inorganic–organic composite modification	/	/	Nanoporous VO_2_ microparticles are embedded inside liquid-crystal matrix for infrared heat-blocking and visibility tuning	[116]
Barinova et al.	Inorganic–organic composite modification	/	/	Femtosecond-laser patterned VO_2_ nanograting metasurface guides liquid-crystal molecular alignment	[117]
Barinova et al.	Inorganic–organic composite modification	/	/	Metamaterial composite constructed by nanoporous VO_2_ microparticles dispersed within liquid-crystal host	[118]

## 6. Conclusions and Perspectives

This paper focused on the three core performance aspects of VO_2_-based thermochromic smart windows, namely phase transition temperature regulation, color regulation, and optical performance regulation, and systematically summarized the mechanisms, research progress, and existing bottlenecks of key modification strategies. These strategies include elemental doping, interfacial strain engineering, micro- and nanostructure engineering, multilayer film design, and inorganic–organic composite modification. In recent years, research on VO_2_-based smart windows has achieved fruitful progress [119,120,121,122,123]; however, numerous bottlenecks in practical applications continue to hinder their large-scale deployment. Based on the current state of research and practical needs, we identify the core challenges and future directions in this field as follows:

(1) Synergistic regulation of the three core properties remains challenging. Most methods for lowering T_c_ are often accompanied by a decrease in T_lum_ or a reduction in ΔT_sol_. The range of color customization is limited, and color optimization frequently conflicts with phase transition characteristics and optical performance, making it difficult to balance aesthetic requirements with energy-saving efficiency. Furthermore, optical performance optimization is constrained by fabrication uniformity and process costs, posing additional hurdles for large-scale applications. In the future, intelligent design methods such as machine learning [124,125,126,127] can be adopted to study complex systems, including fine-tuned doping, multilayer films, and composites, with the goal of developing regulation schemes that achieve synergistic performance optimization.
(2) Stability needs to be improved. Due to the multivalent nature of vanadium, VO_2_ is susceptible to oxidation into V_2_O_5_ under harsh conditions such as high temperature, high humidity, or ultraviolet irradiation, leading to performance degradation. Although strategies such as composite structures, and core–shell structures [128,129] have shown effectiveness in resisting oxidation, they still require further optimization to avoid compromising the intrinsic properties of the material. Concurrently, appropriate performance evaluation standards need to be established to facilitate the synergistic enhancement of both stability and functionality, thereby ensuring long-term service reliability.
(3) Systematic research is still needed to regulate human skin comfort. Smart architectural windows must not only regulate total solar radiation but also prioritize indoor human comfort. The 1350–2500 nm wavelength range in the solar spectrum is a sensitive region for human skin. Existing VO_2_-based thin films lack sufficient regulation over this range, making it difficult to balance energy-saving requirements with skin comfort needs. In the future, multilayer film structures can be designed to specifically regulate transmittance in the skin-sensitive wavelength range, while establishing a standardized comfort evaluation system.
(4) Low-temperature and flexible fabrication remains a significant challenge. VO_2_ film fabrication largely relies on high-temperature processes, making it difficult to deposit directly onto flexible devices or sensitive substrates, which limits its application in curved architecture, wearable devices, and other fields. The development of new technologies, such as low-temperature synthesis processes and layer-by-layer film transfer, is expected to overcome the bottlenecks in flexible fabrication and drive the development of VO_2_-based smart windows toward flexibility and low-temperature fabrication.
(5) Technologies suitable for low-cost, large-scale production remain to be developed. As performance requirements increase, film structures are becoming more complex. Processes such as micro- and nanostructuring and multilayer film deposition are costly and inefficient, making it difficult to meet the demands of large-scale mass production. Therefore, there is an urgent need to develop low-cost, high-precision technologies [130] suitable for large-scale production to drive the development of VO_2_-based smart windows toward low-cost, low-energy-consumption, and large-area industrialization.
(6) Environmental concerns are becoming increasingly prominent. During the fabrication, use, and recycling of VO_2_-based smart windows, there is a risk that vanadium and its compounds may be released into the environment, posing potential threats to ecological safety and human health. Additionally, in outdoor environments, microorganisms can easily form biofilms on the film surface, eroding the coating and affecting optical performance. Therefore, developing low-toxicity, environmentally friendly material systems and introducing self-cleaning functional layers such as TiO_2_ to enhance surface resistance to biofouling are key directions for promoting the green application of VO_2_-based smart windows in the future.

**Figure 9:**
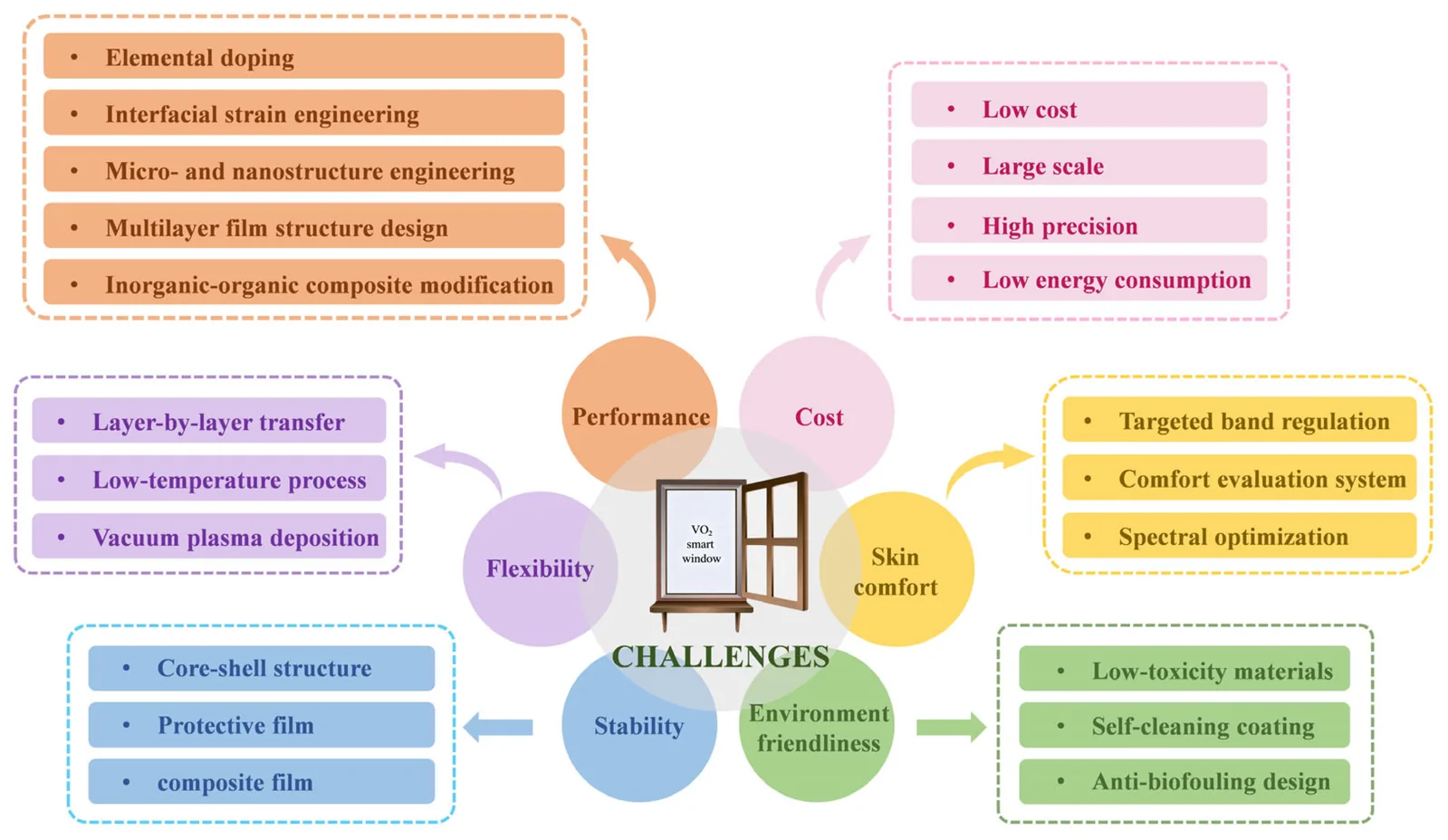
Challenges in the development of VO_2_-based smart windows.

To visually illustrate the development challenges and optimization strategies for VO_2_-based smart windows, we have created Figure 9, which systematically outlines the existing challenges and feasible solutions across various research directions, with the aim of facilitating the advancement of VO_2_-based smart windows toward high performance, multifunctionality, low cost, and scalable application.

## Figures and Tables

**Table 2:** Color regulation in VO_2_-based smart windows.

Authors	Methods	Color	Descriptions	Ref.
Zhou et al.	Mg doping	Significant reduction in yellow hue, increased film brightness	Mg-doped VO_2_ nanoparticles	[76]
Jiang et al.	Zn doping	Marked reduction in brownish-yellow hue	V_1−x_Zn_x_O_2_ film	[77]
Shen et al.	W-Zr co-doping	Yellow hue significantly lightened	W-Zr co-doped VO_2_ nanoparticles	[78]
Christopher et al.	W doping	Transition from brownish-yellow to greenish-blue	W-doped VO_2_ film	[79]
Zhou et al.	Micro- and nanostructure engineering	Dilution of VO_2_ brownish-yellow hue	Periodic array of Ag particles embedded in VO_2_ (M1) thin films	[80]
Shu et al.	Micro- and nanostructure engineering	Customizable visible light hues	Ag nanoarrays on VO_2_ surfaces	[81]
Wei et al.	Micro- and nanostructure engineering	Enables continuous color gradients	Cascaded VO_2_-based Fabry-Pérot optical cavity	[82]
Cheng et al.	Micro- and nanostructure engineering	Lime green at room temperature, turning to yellow-brown after phase transition	Designed MoS_2_/VO_2_/Au multilayer metasurface	[83]
Ji et al.	Optimization of composite systems	Transition from blue-black to gray-green	VO_2_/ZnS core–shell nanoparticles	[84]
Lu et al.	Optimization of composite systems	Capable of displaying various colors such as brick red, purple, and blue	VO_2_@SiO_2_@Au core–shell structures	[85]
Zhu et al.	Optimization of composite systems	Transition from brownish-yellow to dark green upon heating	Composite of VO_2_ nanoparticles and Co^II^-Br-TMP complex	[86]
Zhu et al.	Optimization of composite systems	Transition from light brown to dark green upon heating	Composite of VO_2_ nanoparticles and IL-Ni-Cl complex	[87]
Zhou et al.	Optimization of composite systems	Transparent at low temperatures; becomes semi-translucent brown upon heating	Composite of VO_2_ and PNIPAm hydrogel material	[88]
Hu et al.	Optimization of composite systems	Transition from brownish-yellow to blue-green	VO_2_/Au/VO_2_ composite structure	[89]

## Data Availability

Data sharing is not applicable to this article as no new data were created or analyzed in this study.
